# *Caulophyllum robustum* Maxim in the management of rheumatoid arthritis: a comprehensive review of ethnopharmacology, phytochemistry, pharmacokinetics, and pharmacological mechanisms

**DOI:** 10.3389/fphar.2026.1863086

**Published:** 2026-07-06

**Authors:** Xinyue Yu, Liang He, Guoyu Li, Yuyan Guo, Haixue Kuang, Shaowa Lv

**Affiliations:** 1 Key Laboratory of the Ministry of Education, Department of Pharmacology, Heilongjiang University of Chinese Medicine, Harbin, Heilongjiang, China; 2 School of Pharmacy, Harbin University of Commerce, Harbin, China

**Keywords:** Caulophyllum robustum maxim, ethnopharmacology, pharmacokinetics, pharmacology, phytochemistry, rheumatoid arthritis

## Abstract

**Background:**

Rheumatoid arthritis (RA) is a systemic autoimmune disorder in which persistent synovitis drives progressive joint damage, often leading to functional limitation and a reduced quality of life. *Caulophyllum robustum* Maxim. (*C. robustum*) has long been used in traditional medicine to manage RA. Although increasing experimental evidence has demonstrated its therapeutic potential, relevant studies remain fragmented across various fields.

**Purpose:**

This review aims to systematically integrate current knowledge on the ethnopharmacology, phytochemistry, pharmacokinetics, anti-RA pharmacological mechanisms, and applications of emerging technologies related to *C. robustum*. By synthesizing evidence across multiple disciplines, we seek to clarify its material basis, mechanistic rationale, and therapeutic relevance in RA, thereby providing guidance for future research and drug development.

**Methods:**

To compile evidence on *C. robustum*, we searched multiple databases (Web of Science, PubMed, Google Scholar, Baidu Scholar, and CNKI) and additionally consulted classical Chinese medical texts.

**Results:**

To date, more than 100 compounds have been isolated and identified from *C. robustum*, among which triterpenoid saponins and alkaloids represent the principal bioactive constituents associated with anti-RA activity. Notably, saponin components exhibit interconversion and biotransformation behaviors *in vivo*. Pharmacokinetic studies indicate rapid absorption but relatively slow elimination of key active constituents. Mechanistically, *C. robustum* extracts and representative components exert anti-RA effects by suppressing inflammatory responses, attenuating synovial hyperplasia, inhibiting pannus formation, protecting bone integrity, and restoring immune homeostasis. These actions appear to involve coordinated regulation of several pathways, notably NF-κB, MAPK, JAK/STAT, and PI3K/Akt signaling. In addition, molecular docking, biomolecular interaction analyses, and multi-omics approaches provide further preclinical evidence that saponins and alkaloids from *C. robustum* interact with key targets, including HDACs and CD_81_. Through complementary, multi-target, and network-level regulation, these components collectively contribute to its anti-RA pharmacological activity.

**Conclusion:**

This review provides a comprehensive and integrated overview of *C. robustum*, systematically consolidating research progress across diverse disciplines. By identifying current knowledge gaps and unresolved issues, it highlights critical directions for future investigation. Notably, all current pharmacological evidence for *C. robustum* is drawn exclusively from preclinical studies, with human pharmacokinetic or clinical validation still urgently needed.

## Graphical Abstract

**Figure:**
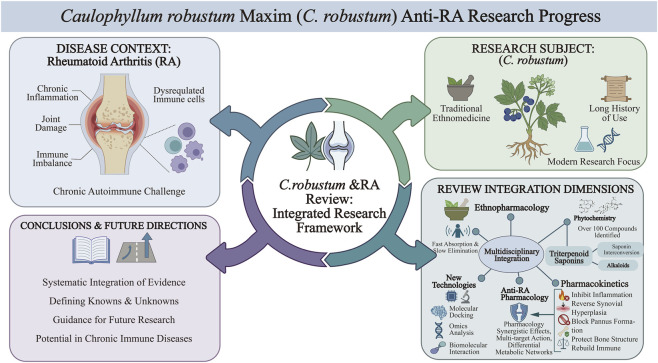

## 1 Introduction

Rheumatoid arthritis (RA) is a chronic systemic autoimmune disease characterized by persistent synovitis and synovial hyperplasia, which progressively erode cartilage and bone (Ma L. et al., 2020; Shakeel et al., 2025). The resulting irreversible structural damage leads to long-term disability and substantially impairs quality of life (Yang et al., 2025). RA occurs worldwide; onset is most frequent between 25 and 55 years of age, and women are affected roughly two to three times more often than men (Zou et al., 2023). Although modern disease-modifying antirheumatic drugs and biological agents have improved disease control, their long-term use is limited by adverse effects, high cost, and inadequate response in many patients (Smolen et al., 2026). These challenges underscore the urgent need for safer, affordable, and more effective therapeutic options (Liao et al., 2025). Historically, natural products—particularly those derived from medicinal plants—have served as a rich source of pharmaceutical agents for chronic diseases, including immune and inflammatory disorders (Butler and La Clair, 2026). Traditional Chinese medicine (TCM), which is fundamentally based on the use of plant-derived remedies, offers over two millennia of clinical experience in managing RA and similar conditions (Wang J. et al., 2025). Unlike single-target pharmacotherapies, TCM emphasizes holistic regulation, aiming to restore systemic balance through multi-component and multi-target interventions (Wang F. F. et al., 2023). In the context of RA, TCM formulations are traditionally used to dispel wind and dampness, promote blood circulation, relieve pain, and modulate immune function, often achieving symptomatic improvement with comparatively fewer adverse effects (Liang et al., 2023). Increasing pharmacological evidence has begun to substantiate these traditional concepts, highlighting the potential of TCM-derived herbs as valuable sources for novel anti-RA agents (Shi et al., 2020; Wang F. F. et al., 2023).

*Caulophyllum robustum* Maxim (*C. robustum*) is a representative TCM herb with a long history of folk application. Its dried roots and rhizomes are commonly used for medicinal purposes and are traditionally described as warm in nature, bitter and pungent in taste, and effective in dispelling wind, unblocking meridians, promoting blood circulation, regulating qi, and alleviating pain (Nanjing University of Chinese Medicine, 2006; Xia et al., 2014b). Historically, *C. robustum* has been widely prescribed for the treatment of rheumatoid conditions, traumatic injuries, and musculoskeletal pain. Authoritative materia medica, including *the Great Dictionary of Chinese Materia Medica*, document its prominent therapeutic effects against arthritis (Institute of Chinese Materia Medica, 1959; Nanjing University of Chinese Medicine, 2006). In recent decades, modern pharmacological studies have provided growing experimental support for the traditional use of *C. robustum*. Extracts of *C. robustum* have been shown to alleviate disease severity in collagen-induced arthritis (CIA) models, accompanied by the suppression of pro-inflammatory mediators, inhibition of synovial hyperplasia, and regulation of immune cell function (Ge et al., 2008; Guo et al., 2020a; Lv et al., 2019). Advanced analytical approaches, including metabolomics and network-based analyses, further suggest that *C. robustum* exerts broad regulatory effects on inflammation-related signaling pathways, immune homeostasis, and bone remodeling processes (Wang et al., 2023d; Zhu et al., 2023). Collectively, these preclinical findings suggest that *C. robustum* may serve as a promising plant-derived candidate for RA management, pending further pharmacokinetic and safety evaluations in humans, followed by clinical trials to validate its therapeutic value.

Despite these advances, current research on *C. robustum* remains scattered across multiple disciplines, and a systematic synthesis is still lacking. The core bioactive constituents responsible for its anti-RA effects, their pharmacokinetic behaviors, and their mechanistic links to modern pharmacological targets have not been comprehensively clarified (Duan et al., 2021b; Lv et al., 2021). Moreover, the relationship between traditional usage, *in vivo* biotransformation, and contemporary drug development paradigms warrants further integration. In this review, we critically appraise research on *C. robustum*, with emphasis on traditional use, chemical constituents, pharmacokinetic features, anti-RA mechanisms, and emerging approaches such as molecular docking and multi-omics. By bridging traditional knowledge with modern biomedical research, this review aims to provide a systematic framework for understanding the therapeutic potential of *C. robustum* and to facilitate its further translational development as a phytomedicine for RA, noting that all current evidence remains at the preclinical stage.

## 2 Materials and methods

This systematic review was conducted and reported in accordance with the Preferred Reporting Items for Systematic Reviews and Meta-Analyses (PRISMA) 2020 statement (Page et al., 2021).

Literature data on the traditional uses, ethnopharmacology, phytochemistry, pharmacokinetics, and anti-RA effects of *C. robustum* were primarily gathered from digital databases, including Web of Science, PubMed, Google Scholar, Baidu Scholar, and CNKI, as well as classical Chinese medical texts. The search incorporated combinations of the following keywords: (“*C. robustum*” OR “Hong Mao Qi” OR “Leontice robusta”) AND (“rheumatoid arthritis” OR “RA” OR “inflammation” OR “immunomodulation” OR “phytochemistry” OR “pharmacokinetics” OR “mechanism” OR “molecular docking” OR “metabolomics” OR “genomics”).

All peer-reviewed journal publications up to 31 January 2026, were reviewed, yielding over 573 relevant records spanning 1959 to 2025. Journal eligibility was based on articles written in English and Chinese. The inclusion criteria were established based on the proposed inquiries outlined in the introduction: i) traditional ethnomedicinal uses of *C. robustum*, ii) phytochemical constituents and structural characterization, iii) pharmacokinetic and metabolic properties, and iv) *in vitro* and *in vivo* anti-RA pharmacological effects and underlying mechanisms. Studies unrelated to the composition, disposition, or anti-RA activities of *C. robustum* were excluded.

**FIGURE 1:**
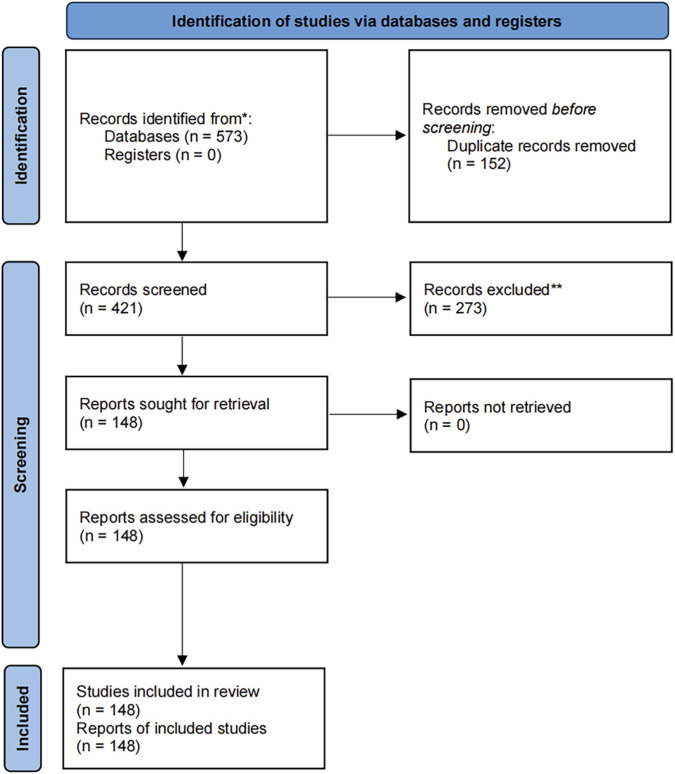
PRISMA 2020 flow diagram of the literature search and selection process.

After removing duplicate records, 148 studies were included for systematic synthesis and analysis. The detailed literature screening process is illustrated in the PRISMA flow diagram (Figure 1). Botanical names were verified using the World Flora Online database (accessed 13 November 2025, at http://www.worldfloraonline.org). Structures were prepared in ChemDraw (v23.1.1, 64-bit), and CAS registry numbers were verified against the PubChem database.

## 3 Botany and botanical identification

**FIGURE 2:**
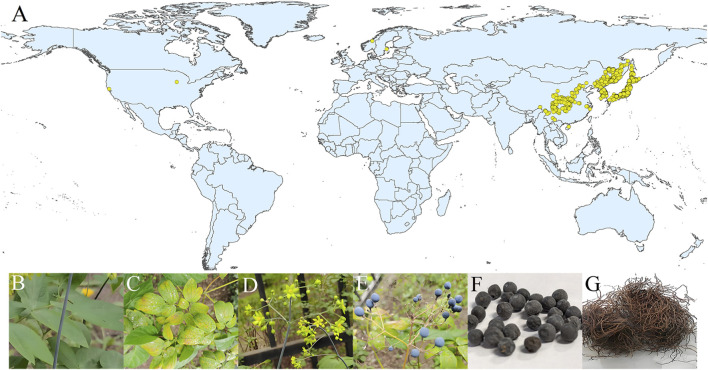
Plant Characteristics and Global Distribution of *C. robustum* around the world. **(A)** The general worldwide distribution of *C. robustum*; **(B)** Stem; **(C)** Leaves; **(D)** Flowers; **(E)** Fruits; **(F)** Seeds; **(G)** Roots and rhizomes.

*Caulophyllum robustum* belongs to the genus *Caulophyllum* within the family Berberidaceae and is a perennial herbaceous plant. In traditional medicine, the dried roots and rhizomes of *C. robustum* are used as the medicinal parts. Accurate botanical identification is essential for medicinal plant research, as taxonomic ambiguity may lead to inconsistency in phytochemical composition, pharmacological activity, and safety evaluation. *Caulophyllum robustum* is widely distributed in China, occurring in multiple provinces including Heilongjiang, Jilin, Liaoning, Hebei, Henan, Hubei, Shaanxi, Shanxi, Sichuan, Yunnan, Zhejiang, Anhui, Guizhou, and Tibet. In addition to its presence in East Asia, the genus *Caulophyllum* is also distributed in North America and parts of Northeast Asia. The general geographic distribution of *C. robustum* is illustrated in Figure 2A, which provides a global overview of its natural occurrence (Data source: GBIF, https://www.gbif.org). Such a broad distribution suggests ecological adaptability, but also underscores the importance of species-level authentication in different regions.

Within the genus *Caulophyllum*, two closely related species, *Caulophyllum thalictroides* (L.) Michx. and *Caulophyllum giganteum* (Farw.) H. Loconte and W. H. Blackwell have also been reported and used medicinally in certain regions, particularly in North America (Sorrie, 2005). Although these species share morphological similarities and partially overlapping chemical profiles, differences in geographic distribution, constituent composition, and reported bioactivities have been documented. Therefore, accurate authentication of *C. robustum* is essential for reliable and comparable experimental results. Morphologically, *C. robustum* can be identified as an erect herb reaching up to approximately 80 cm in height, with thick, short rhizomes and alternately arranged compound leaves. The plant bears terminal inflorescences with small, pale yellowish-green flowers, followed by characteristic blue-black seeds at maturity (College, 1977; Cao, 1998; Nanjing University of Chinese Medicine, 2006). Representative morphological features of the stem, leaves, flowers, fruits, seeds, and underground parts are shown in Figures 2B–G, providing visual references for botanical authentication. From a pharmacognostic perspective, these diagnostic traits offer practical criteria for distinguishing *C. robustum* from related species during field collection and material preparation.

Overall, clarification of botanical identity and geographic distribution provides a necessary foundation for interpreting ethnopharmacological records, phytochemical investigations, and pharmacological studies of *C. robustum*. Establishing this baseline is particularly important for a multi-component phytomedicine, as it ensures consistency, reproducibility, and translational relevance in subsequent research on its anti-RA effects.

## 4 Ethnopharmacology

*Caulophyllum robustum*, also referred to as *Hong Mao Qi* or *Wei Yan Xian*, has been used in traditional Chinese and ethnic medical practices for a long period. Its uses are recorded in several authoritative materia medica sources, such as the *Records of Chinese Materia Medica*, the *National Compendium of Chinese Herbal Drugs*, *Chinese Herbal Medicine*, and the *Great Dictionary of Chinese Materia Medica* (Institute of Chinese Materia Medica, 1959; Quan et al., 1975; Cao, 1998; Nanjing University of Chinese Medicine, 2006). These texts describe the herb as warm in nature with a bitter and pungent taste, and it is traditionally prescribed to dispel wind-dampness, activate blood circulation, unblock meridians, regulate qi, and relieve pain. Clinically, *C. robustum* has been applied to rheumatic complaints, traumatic injuries, and musculoskeletal pain, with particular emphasis on arthritis-related conditions (Nanjing University of Chinese Medicine, 2006).

A notable feature of its ethnopharmacological practice is the frequent use of alcoholic preparations. Classical records recommend soaking approximately 9 g of the roots and rhizomes in about 300 mL of liquor for several days before administration, which is believed to enhance efficacy against arthritic symptoms (Nanjing University of Chinese Medicine, 2006; Wang et al., 2017). From a modern pharmacological perspective, alcoholic extraction may facilitate the dissolution and bioavailability of lipophilic constituents, such as triterpenoid saponins and alkaloids, thereby partially explaining the empirical effectiveness of this preparation method.

*Caulophyllum robustum* also occupies an important position in the ethnomedicine of several Chinese ethnic groups, including the Tujia, Qiang, and Miao communities. Among the Tujia people, medicinal liquors represent a core therapeutic modality. Alcohol is traditionally regarded as warm in nature and capable of promoting blood circulation, dispelling cold, and enhancing the delivery of active compounds. In this context, *C. robustum* is frequently combined with herbs such as *Toddalia asiatica*, *Campsis grandiflora*, *Panax bipinnatifidus*, and *Panax japonicus* to prepare alcoholic formulations for alleviating joint pain, limb numbness, and rheumatic discomfort (Dai et al., 2022). Another commonly used formulation incorporates *C. robustum* with *Aconitum hemsleyanum*, *Piper wallichii*, *Spatholobus sinensis*, and *Eleutherococcus giraldii*, which is administered to treat rheumatic bone diseases and swollen, painful joints (Li et al., 2016).

In Qiang and Miao medicine, *C. robustum* is particularly valued for external application, especially in the treatment of fractures and labor-related injuries. It is frequently included as a key component of traditional “medicinal liquors for labor injuries,” reflecting its recognized role in pain relief, inflammation control, and tissue recovery (Liu Z. X. et al., 2020). Notably, regional pharmacopeial standards, such as the *Hubei Provincial Chinese Materia Medica Quality Standards* (2009 edition), explicitly document the anti-inflammatory, analgesic, and anti-rheumatic activities of *C. robustum* extracts (Ge et al., 2008). Clinical observations further support the ethnopharmacological relevance of *C. robustum*. For example, formulations containing *C. robustum* in combination with *T. asiatica*, *Kadsura heteroclita*, and *Zanthoxylum armatum* have been reported to significantly improve RA-related symptoms, with favorable outcomes observed in large patient cohorts (Deng and Liao, 2007). Although these reports are primarily descriptive, they provide valuable empirical evidence that has guided subsequent experimental and pharmacological investigations.

Collectively, the ethnopharmacological applications of *C. robustum* reveal a consistent therapeutic focus on rheumatic pain, inflammatory disorders, and musculoskeletal injuries, achieved through oral administration, topical use, and alcoholic preparations. These traditional practices not only establish the historical empirical use of *C. robustum* but also offer critical clues for understanding its pharmacological properties and for guiding modern mechanistic and translational research in RA.

## 5 Phytochemistry

### 5.1 Overview of chemical constituents

Phytochemical investigations over the past 2 decades have revealed that *C. robustum* possesses a chemically rich and structurally diverse profile, providing a solid material basis for its multi-component and multi-target pharmacological effects. Constituents reported from the roots and rhizomes exceed one hundred in total, spanning triterpenoid saponins, alkaloids, sterols, phenolics, iridoids, sesquiterpenes, volatile fractions, polysaccharides, fatty acids, and other secondary or derivative metabolites (Huang et al., 2020; Lv et al., 2017a; Xia et al., 2014a).

Among these constituents, triterpenoid saponins represent the predominant chemical class in terms of both abundance and structural diversity, accounting for approximately 7.46% of the dry weight of *C. robustum* (Xia et al., 2015). Alkaloids constitute the second major class of bioactive compounds, occurring at comparatively lower levels but exhibiting broad pharmacological activities relevant to inflammation, immune regulation, and bone metabolism. Other chemical components, including sterols, phenolics, polysaccharides, and minor terpenoids, are generally present at lower abundance and are considered auxiliary constituents that may support or modulate the overall pharmacological profile.

From a functional perspective, the hierarchical organization of chemical constituents in *C. robustum* reflects a typical phytomedicine characterized by a small number of dominant bioactive classes accompanied by multiple minor components. This compositional pattern supports a network-based mode of action, in which major constituents provide primary therapeutic effects while minor components contribute complementary antioxidant, immunomodulatory, or pharmacokinetic-modulating functions. Such a chemical framework is consistent with the traditional multi-component therapeutic concept and provides a rational basis for the observed anti-RA activity of *C. robustum*. In addition to compounds isolated through classical phytochemical approaches, recent high-resolution mass spectrometry and metabolomics studies have further expanded the chemical landscape of *C. robustum* by revealing numerous secondary and derivative metabolites, including modified saponins and alkaloid-related metabolites (Jin et al., 2024; Xia et al., 2016). Although many of these metabolites remain incompletely characterized, their detection underscores the dynamic and complex nature of the chemical system underlying the pharmacological effects of *C. robustum*. Collectively, these findings establish a comprehensive chemical framework that underpins subsequent discussions of structure-activity relationships, pharmacokinetics, and mechanism-based pharmacological studies.

### 5.2 Triterpenoid saponins

Saponins have been extensively investigated in studies of autoimmune disease mechanisms owing to their diverse pharmacological activities, particularly anti-inflammatory and immunomodulatory effects (Qu et al., 2022; Xiong et al., 2025). Among them, triterpenoid saponins are the most characteristic bioactive constituents of *C. robustum*, accounting for approximately 7.46% of its dry weight (Xia et al., 2014b; Xia et al., 2015). To date, 68 triterpenoid saponins have been characterized from the genus *Caulophyllum* (Table 1). Structurally, these saponins consist of two fundamental components: an aglycone (AG) moiety and one or more carbohydrate chains.

**TABLE 1:** Triterpenoid saponins in the genus *Caulophyllum.*

No	Formula	Compounds	AG	R_1_	R_2_	Molecular weight	Sources^a^	Ref.
1	C_35_H_56_O_8_	Cauloside A	I	Ara(p)	H	605	Cr,Ct	Xia et al. (2014b)
2	C_41_H_66_O_13_	Cauloside C	I	Glc (1→2) Ara (p)	H	766	Cr,Ct	Xia et al. (2014b)
3	C_53_H_86_O_22_	Cauloside D	I	Ara(p)	Rha (1→4)Glc (1→6)Glc	1074	Cr,Ct	Xia et al. (2014b)
4	C_59_H_96_O_27_	Cauloside G	I	Glc (1→2)Ara(p)	Rha (1→4)Glc (l→6)Glc	1236	Cr,Ct	Xia et al. (2014b)
5	C_41_H_66_O_12_	Cauloside b	I	Rha (1→2)Ara(p)	H	750	Cr	Xia et al. (2014b)
6	C_46_H_74_O_16_	Cauloside c	I	Ara (1→3)Rha (1→2)Ara(p)	H	882	Cr	Xia et al. (2014b)
7	C_48_H_78_O_18_	Hederagenin-28-*O*-*α*-*L*-rhamnopyranosyl-(1→4)-*β*-*D*-glucopyranosyl (1→6)-*β*-*D*- glucopyranosyl ester	I	H	Rha (1→4)Glc (l→6)Glc	942	Ct	Xia et al. (2014b)
8	C_59_H_96_O_27_	3-*O*-*β*-*D*-glucopyranosyl-(1→3)-*α*-*L*-arabinopyranosylhederagenin 28-*O*-*α*-*L*-rhamnopyranosyl-(1→4)-*β*-*D*-glucopyranosyl (1→6)-*β*-*D*-glucopyranoside	I	Glc (1→3)Ara(p)	Rha (1→4)Glc (l→6)Glc	1236	Ct	Xia et al. (2014b)
9	C_77_H_126_O_41_	Leonticin A	I	Glc (1→2)Ara(p)	Rha (1→4)Glc (l→6)Glc-(l→4)Rha (l→4)Glc (l→6)Glc	1706	Cr	Xia et al. (2014a)
10	C_63_H_106_O_31_	Leonticin E	I	g1c (1→2) Ara(p)	Glc (l→3) Rha (1→4)Glc (1→6)Glc	1382	Cr	Xia et al. (2014a)
11	C_59_H_96_O_26_	Hederacoside C	I	Rha (1→2)Ara(p)	Rha (1→4)G1c (1→6)G1c	1221	Cr	Xia et al. (2014a)
12	C_59_H_96_O_26_	3-*O*-*α*-*L*-rhamnopyranosyl-(1→4)-*α*-*L*-arabinopyranosylhederagenin 28-*O*-*α*-*L*-rhamnopyranosyl-(1→4)-*β*-*D*-glucopyranosyl-(1→6)-*β*-*D*-glucopyranosyl ester	I	Rha (1→4)Ara(p)	Rha (1→4)G1c (1→6)G1c	1221	Cr	Xia et al. (2014a)
13	C_47_H_76_O_18_	3-*O*-*β*-*D*-glucopyranosyl (1→2)-*α*-*L*-arabinopyranosyl-hederagenin-28-*O*-*β*-*D*-glucopyranosyl ester	I	Glc (1→2)Ara(p)	Glc(p)	928	Cr	Xia et al. (2014a)
14	C_47_H_76_O_18_	Durupcoside C	I	Glc (1→3) Glc (1→3)Ara(p)	H	928	Cr	Xia et al. (2014a)
15	C_41_H_66_O_13_	3-*O*-*β*-*D*-glucopyranosyl-(1→3)-*α*-*L*-arabinopyranosyl-hederagenin	I	Glc (1→3) Ara(p)	H	766	Ct	Matsuo et al. (2009)
16	C_40_H_64_O_12_	Akeboside La	I	Ara (1→2)Ara(p)	H	736	Cr	Xia et al. (2014a)
17	C_30_H_48_O_4_	Hederagenin	I	H	H	473	Cr	Qin et al. (2018)
18	C_49_H_80_O_20_	HN-saponin F	I	Ara(p)	Glc(p)	989	Cr	Zhang et al. (2025)
19	C_65_H_106_O_32_	Kalopanax saponin G	I	H	Rha (1→4)Glc (1→6)-Glc	1398	Cr	Zhang et al. (2025)
20	C_35_H_56_O_9_	Cauloside B	II	Ara(p)	H	620	Cr,Ct	Xia et al. (2014b)
21	C_53_H_86_O_23_	Leonticin D	II	Ara(p)	Rha (l→4)Glc (1→6)Glc	1090	Cr,Ct	Xia et al. (2014b)
22	C_59_H_96_O_28_	Cauloside H	II	Glc (1→2) Ara(p)	Rha (1→4)Glc (1→6)Glc	1252	Ct	Ali and Khan (2008)
23	C_47_H_76_O_19_	Leiyemudanoside A	II	Ara(p)	Glc (1→6) Glc	944	Cr	Xia et al. (2014b)
24	C_59_H_96_O_28_	Leiyemudanoside B	II	Glc (1→3) Ara(p)	Rha (1→4) Glc (1→6) Glc	1252	Cr	Xia et al. (2014b)
25	C_41_H_66_O_14_	3*β*-[(*O*-*β*-*D*-glucopyranosyl-(1→2)-*α*-*L*-arabinopyranosyl)oxy]-16*α*,23-dihydroxyolean-12-en-28-oic acid	II	Glc (1→2)Ara(p)	H	782	Ct	Matsuo et al. (2009)
26	C_41_H_66_O_14_	3*β*-[(*α*-*L*-arabinopyranosyl)oxy]-16*α*,23-dihydroxyolean-12-en-28-oic acid *β*-*D*-glucopyranosyl ester	II	Ara(p)	Glc	782	Ct	Matsuo et al. (2009)
27	C_59_H_96_O_27_	3-*O*-*α*-*L*-rhamnopyranosyl-(1→2)-*α*-*L*-arabinopyranosyl-caulophyllogenin-28-O-*α*-*L*-rhamnopyranosyl-(1→4)-*β*-*D*-glucopyranosyl-(1→6)-*β*-*D*-glucopyranosyl ester	II	Rha (1→2)Ara(p)	Rha (1→4)Glc (1→6)-Glc	1237	Cr	Xia et al. (2014a)
28	C_42_H_68_O_14_	Congmuyanoside A	II	Glc (1→3)Ara(p)	H	796	Cr	Xia et al. (2014a)
29	C_35_H_56_O_9_	collinsonin	II	H	H	620	Cr	Qin et al. (2018)
30	C_56_H_88_O_27_	C.Spanion A	II	H	Rha (1→4)Glc (1→6)-Glc	1193	Cr	Zhang et al. (2025)
31	C_71_H_116_O_37_	Leiyemudanoside D	II	Ara(p)	Glc (1→4) Glc (1→6)Rha (1→4)Rha (1→4) Glc (1→6)Glc	1560	Cr	Xia et al. (2014b)
32	C_35_H_56_O_7_	3*β*-[(*α*-*L*-arabinopyranosyl) oxy] olean-12-en-28-oic acid	III	Ara(p)	H	588	Ct	Xia et al. (2014b)
33	C_41_H_66_O_12_	Saponin PE	III	Glc (1→2)Ara(p)	H	750	Ct	Xia et al. (2014b)
34	C_59_H_96_O_26_	Ciwujianoside A	III	Glc (1→2)Ara(p)	Rha (1→4)Glc (1→6)G1c(p)	1221	Ct	Xia et al. (2014b)
35	C_65_H_106_O_31_	Raddeanoside R_9_	III	Glc (1→4)Ara(p)	Rha (1→4)Glc (1→6)G1c(p)	1382	Cr	Xia et al. (2014a)
36	C_55_H_88_O_23_	3-*O*-*β*-*D*-Methylglucuronosyl-oleanolic acid-28-*O*-*α*-*L*-rhamnopyranosyl-(1→4)-*β*-*D*-glucopyranosyl-(1→6)-*β*-*D*-glucopyranosyl	III	GlcUAOCH_3_	Rha (1→4)Glc (1→6)G1c(p)	1117	Cr	Xia et al. (2014a)
37	C_53_H_86_O_21_	Ciwujianoside C3	III	Ara(p)	Rha (1→4)Glc (1→6)G1c(p)	1059	Cr	Xia et al. (2014a)
38	C_41_H_66_O_12_	Guaianin N	III	Glc (1→3)Ara(p)	H	750	Cr	Xia et al. (2014a)
39	C_53_H_86_O_22_	3-*O*-*α*-*L*-arabinopyranosyl-echinocystic acid 28-*O*-*α*-*L*-rhamnopyranosyl-(1→4)-*β*-*D*-glucopyranosyl-(1→6)-*β*-*D*-glucopyranosyl ester	IV	Ara(p)	Rha (1→4)Glc (1→6)Glc	1074	Cr	Xia et al. (2014b)
40	C_41_H_66_O_13_	3-*O*-*β*-*D*-glucopyranosyl-(1→2)-*α*-*L*-arabinopyranosyl-echinocystic acid	IV	Glc (1→2)Ara(p)	H	767	Ct	Xia et al. (2014b)
41	C_59_H_96_O_27_	Leiyemudanoside C	IV	Glc (1→3) Ara(p)	Rha (1→4) Glc (1→6) Glc	1252	Cr	Xia et al. (2014b)
42	C_47_H_76_O_19_	3-*O*-*α*-*L*-arabinopyranosyl-echinocystic acid 28-*O*-*β*-*D*-glucopyranosyl-(1→6)-*β*-*D*-glucopyranosyl ester	IV	Ara(p)	Glc (1→6) Glc(p)	928	Ct	Xia et al. (2014b)
43	C_59_H_96_O_27_	Leiyemudanoside E	IV	Glc (1→2)Ara(p)	Rha (1→4)Glc (1→6)Glc	1236	Cr	Xia et al. (2014b)
44	C_41_H_66_O_13_	3-*O*-*β*-*D*-glucopyranosyl-(1→3)-*α*-*L*-arabinopyranosyl-echinocystic acid	IV	Glc (1→3)Ara(p)	H	767	Cr	Xia et al. (2014a)
45	C_35_H_56_O_8_	3-*O*-*α*-*L*-arabinopyranoside echinocystic acid	IV	Ara(p)	H	604	Cr	Qin et al. (2018)
46	C_48_H_78_O_18_	echinocystic acid-28-*O*-*α*-*L*-rhamnopyranosyl-(1→4)-*β*-*D*-glucopyranosyl-(1→6)-*β*-*D*-glucopyranosyl ester	IV	H	Rha (1→4) Glc (1→6) Glc	942	Cr	Zhang et al. (2025)
47	C_53_H_84_O_23_	Leiyemudanoside G	VI	Ara(p)	G1c (1→4) G1c (1→6)Rha	1088	Cr,Ct	Xia et al. (2014b)
48	C_53_H_84_O_24_	Leiyemudanoside F	VII	Ara(p)	Glc (1→4)Glc (1→6)Rha	1104	Ct	Xia et al. (2014b)
49	C_35_H_54_O_8_	3-*O*-*α*-*L*-arabinopyranosyl-gypsogenin	VIII	Ara(p)	H	602	Ct	Xia et al. (2014b)
50	C_53_H_84_O_23_	3-*O*-*α*-*L*-arabinopyranosyl-gypsogenin-28-*O*-*α*-*L*-rhamnopyranosyl-(1–4)-*β*-*D*-glucopyranosyl-(1–6)-*β*-*D*-glucopyranosyl ester	VIII	Ara(p)	Rha (1→4)Glc (1→6)Glc	1072	Cr	Qin et al. (2018)
51	C_47_H_76_O_20_	3-*O*-*β*-*D*-glucopyranosyl-gypsogenin-28-*O*-*α*-*L*-arabinopyranosyl-(1–6)-*β*-*D*-glucopyranosyl ester	VIII	Glc	Ara (1→6)Glc	960	Ct	Qin et al. (2018)
52	C_59_H_94_O_27_	C. Spanion B	VIII	Glc (1→2)Ara(p)	Rha (1→4)Glc (1→6)Glc	1234	Cr	Zhang et al. (2025)
53	C_35_H_58_O_7_	3*β*-[(*α*-*L*-arabinopyranosyl)oxy]-olean-12-en-28-ol	IX	Ara(p)	-	590	Cr	Xia et al. (2014b)
54	C_35_H_58_O_8_	16*α*,23,28-Trihydroxy-olean-12-en-3-*O*-*α*-*L*-arabinopyranoside	IX	Ara(p)	-	606	Cr	Ma et al. (2012)
55	C_59_H_96_O_26_	3-*O*-*α*-*L*-glucopyranosyl-(1→2)-*α*-*L*-arabinopyranosyl-betulinic acid 28-*O*-*α*-*L*-rhamnopyranosyl-(1→4)-*β*-*D*-glucopyranosyl-(1→6)-*β*-*D*-glucopyranosyl ester	X	Glc (1→2)Ara(p)	Rha (1→4)Glc (1→6)Glc	1220	Cr	Zhang et al. (2025)
56	C_41_H_64_O_14_	3-*O*-*β*-*D*-glucopyranosyl-(1→2)-*α*-*L*-arabinopyranosyl-11-oxo-hederagenin	VI	Glc (1→2) Ara(p)	-	780	Ct	Xia et al. (2014b)
57	C_35_H_54_O_10_	3-*O*-*α*-*L*-arabinopyranosyl-11-oxo-caulophyllogenin	VII	Ara(p)	H	634	Ct	Xia et al. (2014b)
58	C_30_H_44_O_5_	23-hydroxy-3,19-dioxoolean-12-en-28-oic-acid	-	-	-	484	Cr	Qin et al. (2018)
59	C_30_H_44_O_5_	23-hydroxy-3,11-dioxo-olean-12-en-28-oic acid	-	-	-	484	Cr	Qin et al. (2018)
60	C_30_H_44_O_5_	16*α*,23-dihydroxy-3-oxo-olean-12-en-28-oic acid	-	-	-	484	Cr	Qin et al. (2018)
61	C_29_H_47_O_3_	3*β*, 23-dihydroxy-28-norolean-12-ene-16-one	-	-	-	443	Cr	Jin et al. (2024)
62	C_30_H_47_O_5_	3-Oxo-21*β*,23-dihydroxyolean-12-en-28-oic acid	-	-	-	487	Cr	Jin et al. (2024)
63	C_35_H_56_O_8_	3-*O*-*α*-*L*-Arabinopyranosyl-16-*α*-hydroxyolean-12-en-28-oic acid	-	-	-	605	Cr	Ma et al. (2012)
64	C_35_H_54_O_9_	3*β*-[(*α*-*L*-arabinopyranosyl)oxy]-16*α*-hydroxy-23-oxo-olean-12-en-28-oic acid	​	​	​	618	Ct	Xia et al. (2014b)
65	C_35_H_56_O_8_	3*β*-[(*α*-*L*-arabinopyranosyl)oxy]-16*α*,23-dihydroxyoleana-11,13-dien-28-oic acid	-	-	-	605	Cr,Ct	Xia et al. (2014b)
66	C_54_H_88_O_23_	16*α*-*O*-methyl-*3*-*O*-*α*-*L*-arabinopyranosyl-hederagenin-28-*O*-*α*-*L*-rhamnopyranosyl-(1→4)-*β*-*D*-glucopyranosyl-(1→6)-*β*-*D*-glucopyranosyl ester	-	-	-	1104	Cr	Huang et al. (2020)
67	C_41_H_68_O_13_	3*β*-[(*O*-*β*-*D*-glucopyranosyl-(1→2)-*α*-*L*-arabinopyranosyl) oxy]-20*α*-hydroxyursan-28-oic acid	-	-	-	768	Ct	Xia et al. (2014b)
68	C_41_H_66_O_12_	3*β*-[(*O*-*β*-*D*-glucopyranosyl-(1→2)-*α*-*L*-arabinopyranosyl) oxy]-20*β*-hydroxyursan-28-oic acid	-	-	-	750	Ct	Xia et al. (2014b)

^a^ Cr means *C. robustum*; Ct means *C.thalictroides*.

**FIGURE 3:**
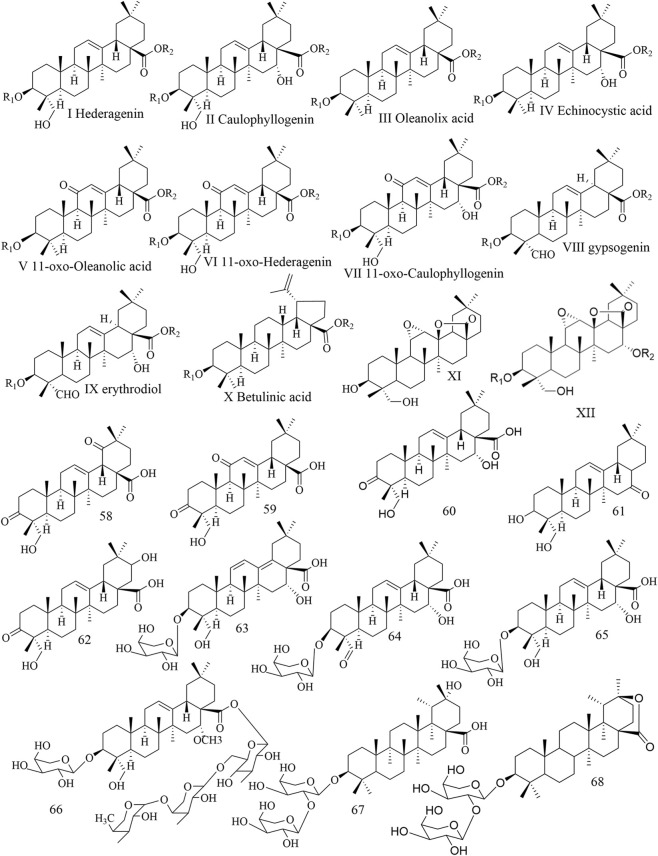
AG and structures of compounds 58–68 from triterpenoid saponins in the genus *Caulophyllum*. (I-XII) AG. (58–68) structures of compounds 58–68.

The AG backbones are predominantly pentacyclic triterpenes, including hederagenin, caulophyllogenin, oleanolic acid, echinocystic acid, 11-oxo-oleanolic acid, 11-oxo-hederagenin, 11-oxo-caulophyllogenin, gypsogenin, erythrodiol, betulinic acid, etc (Figures 3I–X). Through integrated approaches combining network pharmacology and experimental validation, several triterpenoid saponins with anti-RA activity and their underlying mechanisms have been identified. For example, hederagenin strengthens antioxidant defenses by engaging the Nrf2/HO-1 axis, which helps protect chondrocytes from oxidative injury. In addition, it modulates the mitogen-activated protein kinase (MAPK) signaling pathway to suppress proliferation, angiogenesis, and inflammatory responses in RA fibroblast-like synoviocytes (FLS) (Wang et al., 2023c). Oleanolic acid exhibits broad-spectrum anti-inflammatory and immunomodulatory activities (Wu and Yue, 2024). Echinocystic acid suppresses osteoclastogenesis by dampening NF-κB and ERK signaling, thereby alleviating bone erosion (Cheng Y. et al., 2022; Lv et al., 2025).

The carbohydrate chains of triterpenoid saponins are primarily composed of D-glucose (Glc), L-arabinose (Ara), and L-rhamnose (Rha), linked through α- or β-glycosidic bonds. According to their glycosylation patterns, these saponins can be classified into monodesmosidic saponins (MonoS) and bidesmosidic saponins (BideS). MonoS possess carbohydrate chains attached to either the C-3 or C-28 position of the AG, whereas BideS contain carbohydrate chains linked to both the C-3 and C-28 positions (Ali and Khan, 2008; Li et al., 2010). The length, composition, and linkage pattern of the carbohydrate chains critically influence the spatial conformation, solubility, and biological target affinity of saponin molecules, thereby forming the structural basis for their multi-target and broad-spectrum biological activities (Zhu et al., 2020).

Several saponins present at relatively high concentrations or exhibiting notable biological activities in *C. robustum* include Cauloside C (CLC), Cauloside D (CLD), Cauloside G (CLG), Cauloside H (CLH), and Leonticin D (LD). Among these, Cauloside A (CLA) and CLC are classified as MonoS. At 500 and 100 μg/mL, respectively, CLA and CLC reduced root elongation of *Cucumis sativus* L. seedling by 10.3% and 20.4% (Anisimov and Chaikina, 2015). Beyond their phytotoxic effects, CLA and CLC are regarded as core contributors to the anti-RA activity of *C. robustum*. These compounds show antiproliferative activity, at least in part by lowering FAK phosphorylation and subsequently modulating the PI3K/AKT cascade, suggesting potential relevance to autoimmune conditions (Cui M. H. et al., 2025). CLC has also been reported to enhance cell membrane permeability under neutral pH conditions and to inhibit fatty acid synthesis in both *in vitro* and *in vivo* models, properties that may contribute to its biological activity (Anisimov et al., 1978; Li et al., 2018). In contrast, CLD, CLG, and CLH belong to the BideS category. At concentrations of 100 μg/mL for CLD and 250 μg/mL for CLG, these compounds were found to exhibit plant growth–promoting activity (Anisimov and Chaikina, 2015). Notably, CLG can be metabolically converted to CLC *in vivo*, leading to elevated circulating CLC levels and a prolonged elimination half-life. The coexistence of CLG and CLC therefore results in synergistic effects (Li et al., 2018). CLH is characterized by a complex pentasaccharide chain, which may be associated with its pronounced anti-inflammatory activity. In addition, compounds 60 and 61 have been shown to ameliorate insulin resistance and exhibit significant inhibitory activities against protein tyrosine phosphatase 1B and α-glucosidase, indicating broader metabolic regulatory potential (Jin et al., 2024).

### 5.3 Alkaloids

**FIGURE 4:**
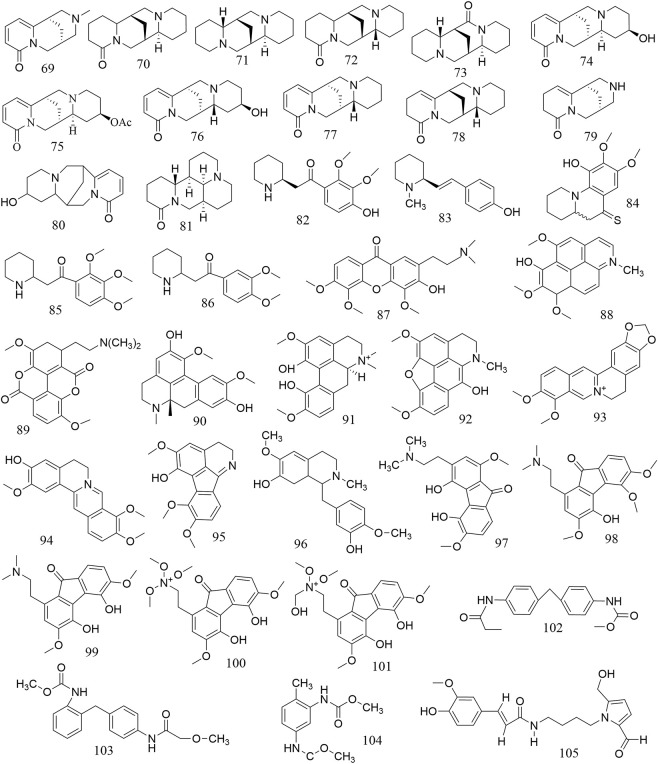
Structure of alkaloid in the genus *Caulophyllum.*

Alkaloids constitute another major bioactive category in *C. robustum* and likely complement the anti-RA actions attributed to triterpenoid saponins. To date, at least 37 alkaloids have been reported from the genus *Caulophyllum*, predominantly from the genus *Caulophyllum* (Table 2), and representative structures are summarized in Figure 4. These alkaloids exhibit substantial structural diversity, encompassing pyridine, quinolizidine, isoquinoline, furanone, and organic amine alkaloids, which collectively underpin their broad pharmacological spectrum.

**TABLE 2:** Alkaloids in the genus *Caulophyllum.*

No	Compounds	Formula	Molecular weight	Sources^a^	Ref.
69	*N*-methylcytisine	C_12_H_16_N_2_O	204	Ct	Avula et al. (2011)
70	Lupanine	C_15_H_24_N_2_O	248	Cr	Yang et al. (2019)
71	Sparteine	C_15_H_26_N_2_	234	Ct	Xia et al. (2014b)
72	*α*-isolupanine	C_15_H_24_N_2_O	248	Cr,Ct	Xia et al. (2014b)
73	Epiaphylline	C_15_H_22_N_2_O_2_	262	Cr	Yang et al. (2019)
74	Pseudohydroxylupanine	C_15_H_24_N_2_O_2_	264	Cr	Yang et al. (2019)
75	*O*-acetylbaptifolin	C_17_H_22_N_2_O_3_	302	Ct	Xia et al. (2014b)
76	Baptifoline	C_15_H_20_N_2_O_2_	244	Cr	Li et al. (2007)
77	Anagyrine	C_15_H_20_N_2_O	244	Cr	Li et al. (2007)
78	5,6-dehydro-*α*-isolupanine	C_15_H_22_N_2_O	246	Cr	Li et al. (2007)
79	Cytisine	C_11_H_14_N_2_O	190	Cr	Xia et al. (2014b)
80	(−)-epimediphine	C_15_H_20_N_2_O_2_	260	Cr	Huang et al. (2020) Wang et al. (2016)
81	Matrine	C_15_H_24_N_2_O	248	Cr	Wei et al. (2022)
82	Caulophyllumines A	C_15_H_21_NO_4_	279	Ct	Ali and Khan (2008)
83	Caulophyllumines B	C_14_H_19_NO	217	Cr	Qin et al. (2020)
84	(±)-caulophine A	C_16_H_21_NO_4_	291	Cr	Yang et al. (2019)
85	(±)-caulophine B	C_16_H_23_NO_4_	293	Cr	Yang et al. (2019)
86	Piperidylacetophenone	C_15_H_21_NO_3_	263	Ct	Ali and Khan (2008)
87	2-(2-(dimethylamino) ethyl)-3-hydroxy-4,5,6-trimethoxy-9H-xanthen-9-one	C_20_H_23_NO_6_	373	Cr	Yang et al. (2019)
88	Caulophyine A	C_19_H_18_NO_4_	324	Cr	Wei et al. (2022)
89	Taspine	C_20_H_19_NO_6_	369	Cr,Ct	Xia et al. (2014b)
90	Boldine	C_19_H_21_NO_4_	327	Cr	Xia et al. (2014b)
91	Magnoflorine	C_20_H_24_NO_4_	342	Cr,Ct	Xia et al. (2014b)
92	Epimediphine	C_21_H_25_NO_4_	355	Cr	Qin et al. (2020)
93	Berberine	C_20_H_18_NO_4_	336	Cr	Park et al. (2023)
94	columbamine	C_20_H_20_NO_4_	338	Cr	Huang et al. (2020)
95	Caulophylline E	C_18_H_17_NO_5_	339	Cr	Li et al. (2007) Wang et al. (2011)
96	(+)-reticuline	C_19_H_23_NO_4_	329	Cr	Huang et al. (2020)
97	Caulophine	C_18_H_15_NO_4_	309	Cr	Li et al. (2007) Wang et al. (2009)
98	Caulophylline A	C_20_H_23_NO_5_	357	Cr	Wang et al. (2011)
99	Caulophylline B	C_19_H_21_NO_5_	343	Cr	Wang et al. (2011)
100	Caulophylline C	C_20_H_24_NO_5_	358	Cr	Wang et al. (2011)
101	Caulophylline D	C_20_H_24_NO_6_	374	Cr	Wang et al. (2011)
102	4,4′-Diphenylmethane-bis(methyl) Carbamate	C_17_H_18_N_2_O_4_	314	Cr	Qin et al. (2019)
103	2,4′-Diphenylmethane-bis(methyl) Carbamate	C_17_H_18_N_2_O_4_	314	Cr	Qin et al. (2019)
104	Obtucarbamate A	C_11_H_12_N_2_O_4_	236	Cr	Ma et al. (2020b) Huang et al. (2020)
105	Magnolamide	C_17_H_19_NO_3_	285	Cr	Qin et al. (2019)

Within the pyridine/quinolizidine subset, cytisine, lupanine, sparteine, and matrine have been associated with analgesic, anti-inflammatory, and immunomodulatory activities (Ali and Khan, 2008; Lv et al., 2019; Zhao et al., 2017). Matrine, in particular, has attracted considerable attention due to its capacity to suppress pro-inflammatory cytokine production, regulate immune cell activation, and ameliorate arthritis symptoms in experimental RA models (Cheng Y. et al., 2022; Zhao et al., 2017). These findings suggest that pyridine-type alkaloids may contribute to symptom relief and immune regulation in RA, complementing the anti-proliferative effects of saponins on synovial tissues.

Isoquinoline alkaloids appear to be the most abundant subclass reported in *C. robustum*. Magnoflorine, often considered a predominant member, displays anti-inflammatory and neuromodulatory properties and may influence cytokine release as well as immune responses (Cafferata et al., 2021). Other isoquinoline alkaloids, including boldine, berberine, and taspine, demonstrate pharmacological activities highly relevant to RA pathology. Notably, boldine has been linked to suppression of osteoclast differentiation and to restoration of Th17/Treg balance, which may confer protection against inflammation-driven bone erosion (Apaza Ticona et al., 2021). Berberine displays well-established anti-inflammatory and metabolic regulatory activities (Sancha et al., 2023). Taspine has also been reported to exert anti-inflammatory effects while promoting tissue repair (Guo et al., 2020a). Collectively, these isoquinoline alkaloids may contribute to both immunomodulatory and bone-protective actions in RA.

Beyond their direct pharmacological effects, alkaloids from *C. robustum* may also modulate the pharmacokinetic behavior of coexisting constituents. Emerging evidence indicates that certain alkaloids, such as obtucarbamate A, can inhibit P-glycoprotein (P-gp)-mediated efflux, thereby enhancing intestinal absorption and systemic exposure of triterpenoid saponins (Guo et al., 2020b). This transporter-modulating effect provides a plausible mechanistic basis for the synergistic activity observed in multi-component extracts of *C. robustum*, highlighting the functional interplay between alkaloids and saponins at the level of drug disposition.

In addition, several alkaloids exhibit antioxidant and cytoprotective activities, which may indirectly alleviate oxidative stress and inflammatory damage within the rheumatoid joint microenvironment. For example, caulophylline E displays potent free-radical-scavenging activity (Xia et al., 2014b), while (+)-reticuline has been reported to exert neuroprotective and metabolic regulatory effects (Nattagh-Eshtivani et al., 2022). Although these effects are not specific to RA, they may contribute to the overall therapeutic profile of *C. robustum* by mitigating secondary pathological processes associated with chronic inflammation.

Taken together, alkaloids in *C. robustum* represent a functionally important component of its anti-RA phytochemical framework. Through direct immunomodulatory and anti-inflammatory actions, regulation of bone metabolism, and modulation of saponin pharmacokinetics, alkaloids act synergistically with triterpenoid saponins to support the multi-target and network-based therapeutic effects of *C. robustum* in RA.

### 5.4 Other chemical components and potential compounds

Beyond triterpenoid saponins and alkaloids, accumulating phytochemical work indicates that *C. robustum* contains a broader set of constituents that further enrich its chemical complexity. Reported minor-to-moderate components span sterols, phenolics, iridoids, sesquiterpenes, volatile fractions, polysaccharides, fatty acids, and several structurally distinctive low-abundance metabolites (Huang et al., 2020; Lv et al., 2017a; Xia et al., 2014a). Although present at comparatively low levels, these molecules may provide complementary bioactivity and collectively reinforce the anti-RA potential of *C. robustum.* Sterols such as β-sitosterol and stigmasterol have been detected in the roots and rhizomes (Lv et al., 2017a). These sterols are widely associated with anti-inflammatory and immunoregulatory actions and may also support bone protection. For instance, β-sitosterol has been linked to reduced pro-inflammatory cytokine output and suppression of osteoclast differentiation, whereas stigmasterol shows anti-inflammatory and antioxidant effects that could mitigate joint inflammation and oxidative stress in RA (Nattagh-Eshtivani et al., 2022). Despite their low abundance in *C. robustum*, sterols may contribute to longer-term immunomodulation and skeletal protection. Phenolic constituents, including flavonoids and simple phenolics, represent another functionally relevant fraction. Their strong antioxidant and radical-quenching capacity may indirectly dampen inflammatory injury within the rheumatoid joint microenvironment (Cui Z. X. et al., 2025). Given the recognized contribution of oxidative stress to RA pathogenesis, phenolics may act as supportive agents that complement the anti-inflammatory effects of saponins and alkaloids. Iridoids and sesquiterpenes have also been reported in *C. robustum*, yet systematic evaluation of their bioactivities remains limited (Xia et al., 2014a). Evidence from other medicinal plants suggests that these classes can display anti-inflammatory and immunoregulatory properties, implying potential relevance to RA. However, their specific contributions within *C. robustum* require further experimental validation. Polysaccharides extracted from *C. robustum* have attracted increasing attention due to their immunomodulatory potential. Preliminary studies indicate that plant-derived polysaccharides can regulate macrophage activation, balance Th1/Th2 immune responses, and enhance immune homeostasis (Wei et al., 2024). Although direct evidence linking *C. robustum* polysaccharides to anti-RA effects is currently limited, their presence provides a plausible basis for synergistic immunomodulation in multi-component extracts.

In addition to known constituents, recent high-resolution mass spectrometry and metabolomics studies have identified several putative or previously uncharacterized compounds in *C. robustum*, including novel malonylated triterpenoid saponins and other derivative structures (Table 3) (Jin et al., 2024; Xia et al., 2016). These discoveries expand the chemical space of *C. robustum* and suggest the existence of additional bioactive molecules that may contribute to its pharmacological effects. However, the biological activities and therapeutic relevance of these potential compounds remain largely unexplored. Overall, while saponins and alkaloids likely form the primary material basis for the anti-RA activity of *C. robustum*, other chemical components may play important auxiliary roles by providing antioxidant protection, immunomodulatory support, and synergistic enhancement of therapeutic efficacy. Further studies integrating targeted isolation, bioactivity evaluation, and systems-level analysis are warranted to clarify the contributions of these minor and potential compounds to the overall pharmacological profile of *C. robustum*.

**TABLE 3:** Reported as potential new compounds.in the *C. robustum*.

No.	AG	R_1_	R_2_	Ref.
106	I	Ara-Ara(p)	Rha (1→4)Glc (1→6)Glc	Xia et al. (2014a)
107	I	Rha (1→3)Ara(p)	Rha (1→4)Glc (1→6)Glc	
108	I	Glc→Acetyl-pentose	Rha (1→4)Glc (1→6)Glc	
109	I	2′-O-Acetyl-pentose	Rha (1→4)Glc (1→6)Glc	
110	I	3′-O-Acetyl-pentose	Rha (1→4)Glc (1→6)Glc	
111	I	4′-O-Acetyl-pentose	Rha (1→4)Glc (1→6)Glc	
112	II	Ara(p)	Rha (1→4)Glc (1→6)Glc→Rha (1→4)Glc (1→6)Glc	
113	II	Acetyl-pentose	Rha (1→4)Glc (1→6)Glc	
114	II	Ara(p)→Acetyl-pentose	Rha (1→4)Glc (1→6)Glc	
115	II	Rha	Rha (1→4)Glc (1→6)Glc (1→4) Rha (1→4)Glc (1→6)Glc	
116	IV	H	Rha (1→4)Glc (1→6)Glc	
117	V	Glc→Ara(p)	Rha (1→4)Glc (1→6)Glc	
118	V	Glc	Rha (1→4)Glc (1→6)Glc	
119	V	Glc (1→3)Ara(p)	H	
120	V	Glc (1→2)Ara(p)	H	
121	VI	Ara(p)	Rha (1→4)Glc (1→6)Glc	
122	VII	Rha→Rha→Ara(p)	Rha (1→4)Glc (1→6)Glc	

### 5.5 Secondary metabolites

A defining feature of *C. robustum* saponins is that their pharmacological relevance in RA hinges on cellular metabolic activation: deglycosylation within FLS converts glycosides into aglycones that are more likely to function as direct effectors at the lesion site. Consistent with oral biotransformation as a prerequisite for *in vivo* efficacy, *C. robustum* saponins yield more active AG forms that underpin key anti-RA activities (Lv et al., 2019). This is mechanistically salient because FLS are principal effector cells driving synovial hyperplasia and sustained inflammation in RA (Gu et al., 2025; Nemeth et al., 2022). Accordingly, metabolite mapping in FLS provides a disease-aligned readout of how “parent” saponins are converted into locally active chemical entities.

**FIGURE 5:**
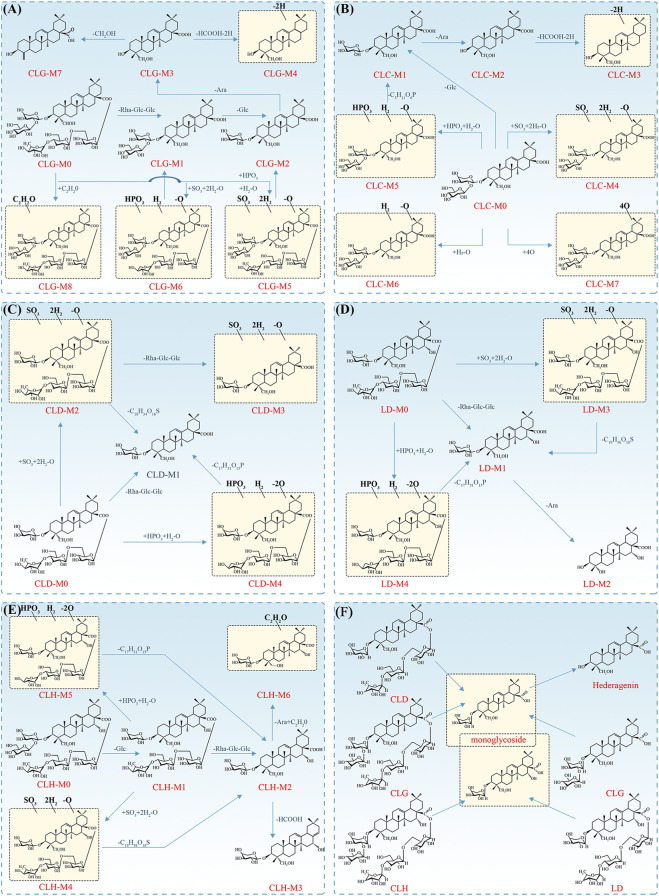
Metabolic behavior of five key saponins from *C. robustum* in FLS and metabolic conversion relationships among the five saponins **(A)** Cauloside G, **(B)** Cauloside C, **(C)** Cauloside D, **(D)** Leonticin D, **(E)** Cauloside H, **(F)** Metabolic conversion relationships among the five saponins. Source: Adapted from Zhu et al. (2023). © The Authors. Adapted with permission. Original figure licensed under CC BY-NC-ND 4.0.

Mass spectrometry–enabled profiling in FLS demonstrates that representative *C. robustum* saponins undergo an organized series of phase I and phase II reactions, with deglycosylation dominating the intracellular fate and dictating the composition of the metabolite pool (Wang et al., 2023d; Zhu et al., 2023). Across structurally distinct precursors, metabolism converges on a restricted set of outcomes, monoglycosides and aglycones, while oxidative/reductive edits and conjugations (e.g., sulfation, phosphorylation, acetylation) diversify and potentially tune local exposure (Wang et al., 2023d; Zhu et al., 2023). Notably, aglycones such as hederagenin and caulophyllogenin have been implicated as joint-accessible effectors capable of directly restraining aberrant FLS proliferation, thereby linking intracellular conversion to plausible anti-synovial hyperplasia activity (Sivakumar et al., 2021). In this framework, the reported two-stage, microenvironment-dependent nature of FLS metabolism becomes conceptually important: it positions the inflamed synovium not merely as a target tissue, but also as a bioreactor that shapes the final effector spectrum (Ma et al., 2024). Figure 5 integrates the key conversion logic and inter-relationships among the major saponins and their metabolite classes (Zhu et al., 2023).

Crucially, these data also establish a coherent structure–metabolism relationship that helps explain why closely related saponins do not yield equivalent effector profiles. Comparative analyses indicate that glycosylation architecture, particularly the number/arrangement of sugar chains at the 3-O position, can determine whether metabolism proceeds to AG formation or stalls at monoglycoside intermediates (Chung et al., 2020; Liu J. H. et al., 2020). In parallel, AG substitution patterns (e.g., hydroxylation differences) appear to bias the depth of deglycosylation, further constraining sapogenin emergence even when sugar chains are similar (Wang L. L. et al., 2023). Together, these observations imply that saponins encode “metabolic instructions” in their scaffolds: glycosylation and AG features jointly dictate whether a given precursor can realistically supply hederagenin- or caulophyllogenin-type effectors within FLS.

The RA microenvironment adds an additional control layer by modulating reaction efficiency and directionality. Elevated β-glucosidase expression in synovial tissues markedly enhances saponin hydrolysis and increases metabolite yield by ∼30–50% (Zhao et al., 2021), providing a lesion-amplified mechanism that would be expected to favor local AG supply. Moreover, inflammatory cues can reprogram cellular metabolic networks in ways that bias product distributions toward anti-inflammatory outputs (Qi et al., 2022). These microenvironmental effects align with target-oriented hypotheses: certain phase I modifications (including dehydration and deacidification) have been proposed to strengthen interactions with RA-relevant targets such as HDAC3, connecting metabolic processing to epigenetic intervention routes (Lv T. T. et al., 2020). In aggregate, current evidence supports the notion that, after oral dosing, systemically generated aglycones can reach the joint via circulation and then either undergo additional FLS-mediated processing or act directly within synovial tissue to suppress inflammation and synovial hyperplasia (Guo et al., 2020a; Lv et al., 2019).

Taken together, secondary metabolism in FLS clarifies the chemical basis of *C. robustum* as a multi-component medicine: parent saponins function as a pro-effector reservoir, while disease-contextual enzymatic and metabolic constraints sculpt a network of locally enriched effectors that can engage inflammatory and epigenetic nodes in parallel. This perspective directly reinforces the central thesis of this review: *C. robustum* exemplifies how complex traditional medicines achieve therapeutic breadth through metabolism-enabled, multi-target pharmacology rather than reliance on a single immutable constituent.

## 6 Pharmacokinetics

Modern pharmacological studies frequently employ *Caulophyllum robustum* Maxim extract (CRME) or its primary active components to systematically evaluate their anti-RA efficacy in parallel with exposure and response considerations. CRME is commonly prepared by reflux extraction of dried roots/rhizomes with 70% ethanol, followed by AB-8 macroporous resin enrichment and collection of the 70% ethanol eluate (Cui M. H. et al., 2025; Guo et al., 2020a). Quantitative profiling shows that CRME is enriched in characteristic triterpenoid saponins, including CLG (28.88%), CLD (10.19%), Cauloside B (10.19%), CLH (6.21%), LD (5.14%), and CLC (2.26%) (Lv et al., 2021). Consistent with phytochemical evidence that triterpenoid saponins and alkaloids represent the principal anti-inflammatory chemical classes in *C. robustum* (Jiang et al., 2022; Liu et al., 2025), multiple UPLC–MS/MS-based studies in rats have delineated the absorption, distribution, metabolism, and excretion (ADME) of CRME and representative saponins/alkaloids.

### 6.1 Absorption

Pharmacokinetic measurements indicate rapid systemic appearance of major saponins (CLC, CLD, CLG, CLH, LD) and the alkaloid magnoflorine in rats, with Tmax values clustered between 0.56 and 1.88 h (Duan et al., 2021b; Guo et al., 2020a). However, intestinal permeability differs markedly by chemical class. In a unidirectional rat intestinal perfusion model, magnoflorine exhibited high effective permeability (Peff) consistent with predominantly passive transport (Guo et al., 2020b). By contrast, saponins showed low permeability (Peff <7.68 × 10^−5^ cm/min), supporting limited intrinsic intestinal uptake (Zhang et al., 2019).

Evidence further indicates that saponin absorption is shaped by anatomical, transporter-related, and structural determinants. First, intestinal segment dependence was observed: CLG, CLH, and LD were preferentially absorbed in the duodenum, whereas CLC and CLD exhibited relatively better absorption in the ileum (Guo et al., 2020b). Potentially reflecting scaffold-dependent interactions with intestinal transport processes (Dubray et al., 2016). Second, permeability displayed minimal concentration dependence (Guo et al., 2020b), implying that uptake is not governed solely by a concentration gradient and may involve carrier-mediated or facilitated components. Third, P-gp efflux materially constrained exposure: verapamil increased Peff for all measured saponins, indicating that these compounds behave as P-gp substrates and that efflux inhibition can enhance oral availability (Dubray et al., 2016; Guo et al., 2020b). Structural features also correlated with absorption kinetics; for example, the disaccharide saponin CLC was absorbed faster than higher-saccharide congeners, consistent with glycosylation burden influencing transmembrane transport efficiency (Guo et al., 2020a). Finally, the rapid appearance of large, polar saponins in plasma may reflect surfactant-like membrane effects (McCalpin et al., 2026) and/or presystemic deglycosylation by gut microbiota/enzes that generates more lipophilic species with improved permeability (Liang et al., 2021; Lv et al., 2019).

Taken together, available data delineate a class-divergent absorption profile: magnoflorine is efficiently absorbed largely via passive diffusion, whereas saponins reach systemic circulation rapidly but with limited permeability and likely constrained oral bioavailability shaped by P-gp efflux, glycosylation architecture, and microbiota-associated conversion.

### 6.2 Distribution

Tissue distribution studies in rats indicate pronounced organ selectivity and temporal variability, providing a tissue-level basis for both therapeutic exposure and off-target burden in highly perfused organs. After oral administration, hepatic concentrations exceeded those in most other tissues (Lv et al., 2019), consistent with the liver’s dual role as a primary metabolic hub and a systemic regulator of inflammatory mediators relevant to RA (Tiegs and Horst, 2022; Zheng et al., 2024). Brain exposure was also measurable: the five quantified saponins reached peak levels at approximately 2.0 h, whereas magnoflorine did not reach an apparent maximum within the sampling window, suggesting more sustained brain distribution for this alkaloid (Duan et al., 2021b), These findings support blood–brain barrier passage for at least part of the chemical repertoire, while the functional implications for central immune modulation warrant direct pharmacodynamic substantiation.

In the heart, quantified constituents peaked early (0.25–0.5 h) and then declined, consistent with perfusion-driven distribution. In the kidney, most components peaked at approximately 0.5 h, whereas CLC peaked later (∼2.0 h) (Liang et al., 2021). Signals were detectable in vascularized peripheral tissues (fat, muscle, and skin), although some measurements approached the detection limit; no corresponding signals were detected in reproductive tissues under the reported conditions (Duan et al., 2021b). Overall, the distribution profile highlights preferential hepatic and renal exposure with measurable central distribution for select constituents, aligning with the dominant biotransformation and elimination pathways described below.

### 6.3 Metabolism

*In vivo* profiling demonstrates extensive biotransformation of CRME constituents following oral administration. Using UPLC–LTQ Orbitrap MS, Lv et al. identified 22 analytes across rat plasma, bile, urine, and feces, including eight parent compounds and 14 metabolites (Lv et al., 2019). The dominant reactions comprised phase I deglycosylation and phase II sulfation/glucuronidation (Lv et al., 2019). Deglycosylation of triterpenoid saponins generated sapogenins (e.g., hederagenin and caulophyllogenin), which subsequently underwent conjugative metabolism in the liver (Lv et al., 2019; Zhu et al., 2023). Matrix-resolved patterns further supported compartment-specific handling: plasma contained both parent compounds and metabolites, whereas bile was dominated by conjugated metabolites (notably sulfates and glucuronides), consistent with efficient hepatic phase II conversion and biliary secretion (Lv et al., 2019). Notably, saponins sharing the same sapogenin scaffold exhibited broadly similar transformation patterns, providing a disposition-level explanation for convergent *in vivo* exposure to overlapping metabolite classes (Yang X. R. et al., 2023).

### 6.4 Excretion

CRME-related constituents were eliminated primarily via urine, with additional contributions from bile and feces. Reported half-lives (t1/2) for major saponins (CLC, CLD, CLG, CLH, LD) ranged from 6.22 to 13.97 h, indicating relatively slow systemic elimination (Guo et al., 2020a).

Urinary profiles contained both sapogenins and downstream metabolites derived from saponins and magnoflorine, supporting partial renal elimination of biotransformation products. In contrast, bile predominantly contained conjugated metabolites with minimal detectable free sapogenins, consistent with hepatic conversion followed by biliary excretion and potential enterohepatic recycling (Yang Z. M. et al., 2023). Fecal samples contained trace parent compounds together with deglycosylated and sulfated species, implicating intestinal processes (including microbiota-associated transformation) in terminal elimination (Lv et al., 2021). Collectively, bile enrichment of conjugates, together with slow elimination kinetics, supports meaningful enterohepatic circulation, which may prolong systemic residence time and should be considered when extrapolating exposure and designing dosing regimens.

Current ADME evidence supports rapid absorption, preferential hepatic/renal distribution, extensive deglycosylation followed by phase II conjugation, and mixed urinary–biliary elimination with potential enterohepatic recycling for key CRME constituents. Nevertheless, quantitative integration across these preclinical studies remains limited; future work should prioritize standardized dosing/analytical platforms, absolute bioavailability estimates for representative saponins and alkaloids, and explicit linking of systemic metabolite exposure to pharmacodynamic readouts in RA models. Ultimately, these preclinical pharmacokinetic findings will need to be validated in human studies to establish clinically relevant parameters.

## 7 Pharmacological effects

*Caulophyllum robustum* exhibits pronounced pharmacological effects against RA, which are attributable not only to its rich repertoire of bioactive constituents but also to the complementary interactions among these components. Previous studies have demonstrated that a combination of total saponins and total alkaloids from *C. robustum* at a ratio of 9:1 produces more pronounced anti-RA effects compared with either fraction alone. In CIA mice, the optimized formulation alleviated clinical signs of arthritis and markedly suppressed a panel of inflammatory mediators, including IL-4, IL-10, IL-17, IFN-γ, granulocyte colony-stimulating factor, and TGF-β. In parallel, it reduced VEGF expression (attenuating pannus angiogenesis) and enhanced bone protection by downregulating RANKL. Concurrently, it promoted apoptosis of abnormally proliferating synovial cells, collectively contributing to the overall anti-RA effects observed in preclinical models (Lv C. Y. et al., 2018).

At the molecular level, this synergistic formulation exerts coordinated regulatory effects on multiple signaling pathways. It limits NF-κB p65 nuclear translocation, shifts the Bcl-2/Bax balance toward apoptosis in synovial cells, increases FasL expression, and suppresses activation of the NF-κB, MAPK, and JAK/STAT pathways. These effects are accompanied by modulation of immune cell function, highlighting the multi-target pharmacological profile of *C. robustum* in RA (Lv C. Y. et al., 2018; Wang et al., 2017). Based on these findings, we stratify all preclinical pharmacological evidence of *C. robustum* against RA into four tiers: (1) crude extract (CRME) studies, (2) enriched fraction (total saponins, total alkaloids, and their combinations) studies, (3) isolated pure compound activities, and (4) *in silico* target predictions. Key experimental findings are summarized in Table 4. Subsequent sections detail the anti-RA pharmacological effects and underlying mechanisms, organized by the core pathological processes of RA.

**TABLE 4:** Pharmacological activities of *C. robustum* against RA.

Evidence tier	Pharmacological activity	Extracts/Compounds	Models/Methods	Dose	Key results	Positive control	Experimental duration	Minimum active dose/IC_50_	Ref.
I. CRME studies	Inhibition of inflammation Reversal of synovial hyperplasia Inhibition of inflammatory cytokine secretion	CRME	CIA mice	24.8–99.4 mg/kg	1. Reduces serum levels of IL-6, TNF-α, PGE_2_ and IL-1β 2. Improves arthritis index and paw swelling 3. Attenuates synovial hyperplasia, capillary dilation and inflammatory cell infiltration	Methotrexate; Tripterygium glycosides	30 days	24.8 mg/kg	Wang et al. (2017)
	Inhibition of inflammation	CRME	LPS-induced RAW264.7 macrophages	0.063–0.250 mg/mL	Inhibits NO production; downregulates mRNA levels of TNF-α, IL-1β and IL-6	-	24 h	0.063 mg/mL	Wang et al. (2017)
	Inhibition of inflammation Reversal of synovial hyperplasia Inhibition of pannus formation Bone protection	CRME	TNF-α-induced MH7A RA-FLS	50–500 μg/mL	1. Reduces IL-6 secretion; elevates IL-4 level 2. Upregulates Bax and FasL; downregulates Bcl-2; promotes synoviocyte apoptosis 3. Decreases VEGF and RANKL expression	-	24 h	50 μg/mL (inflammation/hyperplasia) 100 μg/mL (pannus formation) 500 μg/mL (bone protection)IC_50_ = 645.32 μg/mL (IL-6)	Lv et al. (2018b)
	Inhibition of inflammation	CRME	TNF-α-induced L929 fibroblasts	3.90625–125 μg/mL	Inhibits IL-6 production; regulates 6 key differential genes; involves NF-κB signaling pathway	-	24 h	3.90625 μg/mL	Lv et al. (2020a)
	Bone protection	CRME	CIA mice	33.1–99.4 mg/kg	Decreases RANKL expression; lowers RANKL/OPG ratio; inhibits osteoclastogenesis	Methotrexate	-	33.1 mg/kg	Wang et al. (2017)
	Immunomodulation	CRME	AA rats	17.31–69.23 mg/kg	Reduces spleen/thymus index; regulates T cell subsets; restores Th17/Treg balance	-	-	17.31 mg/kg	Lv et al. (2017b)
	Inhibition of pannus formation	CRME	HUVECs	-	Inhibits endothelial cell proliferation, migration and tube formation; downregulates VEGF, VEGFR2, MMP-2 and MMP-9	-	-	-	Cui et al. (2025a)
​	Inhibition of inflammation Inhibition of JAK/STAT signaling pathway	CRME (joint injection)	Freund’s complete adjuvant-induced rabbit RA model	4.06–16.24 mg/kg	1. Attenuates knee joint swelling and synovial hyperplasia; elevates pain threshold 2. Reduces TNF-α, IL-1β and IL-6 levels in serum and synovial fluid; inhibits JAK2/STAT3 phosphorylation	Lugua polypeptide injection	42 days	4.06 mg/kg	Lv et al. (2025)
II. Enriched fraction studies	Reversal of synovial hyperplasia Inhibition of inflammatory cytokine secretionImmunomodulation	Total saponins + total alkaloids (9:1)	CIA mice	24.86–99.42 mg/kg	1. Decreases Bcl-2/Bax ratio; induces synoviocyte apoptosis 2. Reduces paw swelling degree and arthritis index; lowers serum IL-6 and TNF-α levels 3. Exhibits synergistic anti-inflammatory and immunomodulatory effects compared with single components	Methotrexate Tripterygium glycosides	23 days	24.86 mg/kg	Lv et al. (2018a)
III. Isolated pure compound studies	Inhibition of inflammation	Hederagenin	LPS-induced RAW264.7 macrophages	10–100 μM	Inhibits production of NO, PGE_2_ and pro-inflammatory cytokines; downregulates iNOS and COX-2; suppresses NF-κB pathway	L-NILNS-398	20 h	10 μM	Lee et al. (2015), Zhang et al. (2024)
	Inhibition of inflammation	Hederagenin	Carrageenan-induced mouse paw edema	5–30 mg/kg	Attenuates paw swelling; reduces inflammatory cell infiltration; inhibits mast cell degranulation	Dexamethasone	4 days	30 mg/kg	Lee et al. (2015), Zhang et al. (2024)
​	Inhibition of inflammation Inhibition of synovial cell proliferation and invasion Inhibition of JAK/STAT signaling pathway Inhibition of pannus formation	Hederagenin	IL-1β-induced human RA-FLS	1–50 μM	1. Inhibits secretion of IL-6, IL-8 and CXCL10 2. Inhibits RA-FLS proliferation, migration and invasion; targets JAK2/STAT 3 axis3. Blocks IL-6-induced STAT3 nuclear translocation 4. Inhibits tube formation; reduces VEGF secretion; suppresses MAPK pathway phosphorylation	Tofacitinib	24 h	1 μM (proliferation/JAK/STAT)25 μM (inflammation/pannus formation)25 μM (migration/invasion)	Cheng et al. (2022b), Wang et al. (2023c), Zhang et al. (2024)
	Inhibition of HDAC activity	Hederagenin, Echinocystic acid, Oleanolic acid	*In vitro* HDAC3/8 enzyme activity assay	0.5–500 μM	Dose-dependently inhibits HDAC3 and HDAC8 activity	TSA	-	0.5 μM	Zhao et al. (2020)
	Inhibition of JAK/STAT signaling pathway	Echinocystic acid	IL-1β-induced human RA-FLS	1–50 μM	Blocks IL-6-induced STAT3 nuclear translocation	Tofacitinib	24 h	1 μM	Cheng et al. (2022b)
	Bone protection	Echinocystic acid	RANKL-induced osteoclast differentiation model	10–40 μM	Inhibits osteoclast formation and bone resorption; attenuates joint bone erosion in SKG mice	-	-	10 μM	Cheng et al. (2022b)
IV. In silico prediction studies	Anti-inflammatory target prediction	26 alkaloid components	Molecular docking	-	Screened 9 compounds with good binding activity; mainly targets MIF, COX-2, p38 MAPKetc.	-	-	Docking score ≥7	Yuan (2021)
	Core anti-RA component prediction	15 saponin components	Network pharmacology + molecular docking	-	Predicted echinocystic acid, oleanolic acid and hederagenin as core components; involves JAK/STAT pathway	-	-	-	Lv et al. (2025)
	HDAC target prediction	11 saponins and sapogenins	Molecular docking	-	Hederagenin, echinocystic acid and oleanolic acid show strong binding affinity to HDAC3/8	TSA	-	Binding energy < −24 kJ/mol	Zhao et al. (2020)
	Differential gene regulation prediction	CRME	lncRNA-mRNA microarray analysis	-	Identified 409 differentially expressed genes; constructed lncRNA-mRNA co-expression network	-	-	-	Lv et al. (2020a)

### 7.1 Suppressing inflammatory responses in RA

In RA, dysregulated inflammatory signaling is a key driver of disease progression. Aberrant activity of pathways such as NF-κB, MAPK, and the JAK/STAT cascade promotes the release of a broad spectrum of pro-inflammatory mediators (e.g., TNF-α, IL-6, IL-1β, IFN-γ, and IL-17), together with inflammatory effectors including PGE_2_ and nitric oxide. This sustained mediator milieu contributes to synovitis and, over time, accelerates bone erosion and structural joint damage (Granet et al., 2004; Komatsu and Takayanagi, 2022; Kour et al., 2022; Lv et al., 2017b). Accumulating evidence indicates that CRME effectively modulates the production of pro-inflammatory mediators and alleviates arthritis symptoms. Notably, its pharmacological activity has been reported to surpass that of methotrexate and Tripterygium glycosides in preclinical experimental models, highlighting its potential advantages in the development of safer and more effective anti-RA therapies.

Early pharmacological studies showed that decoctions of *C. robustum* significantly inhibit granulation tissue proliferation and capillary permeability in rats, exerting inhibitory effects on both the exudative and proliferative phases of inflammation (Jiao et al., 1997). Subsequent studies further support that CRME counteracts RA-related pathology through convergent mechanisms, dampening inflammatory responses, promoting synovial-cell apoptosis, and modulating both cellular and humoral immune functions (Lv S. W. et al., 2020; Lv et al., 2017b; Wang et al., 2017).

CRME exhibits pronounced anti-inflammatory activity in both cellular assays and animal models. In LPS-challenged RAW264.7 macrophages, it dose-dependently decreased NO release and lowered mRNA levels of IL-1β, TNF-α, and IL-6 (Wang et al., 2017). In TNF-α-induced MH7A synovial cells, CRME suppresses IL-6 secretion while promoting the release of the anti-inflammatory cytokine IL-4, suggesting a bidirectional regulatory effect on inflammatory balance (Lv S. W. et al., 2018). Moreover, in synovial cells from patients with RA, CRME reduced the expression of NF-κB p50/p65 and IκBα (Zeng et al., 2021).

Consistent with these *in vitro* findings, CRME significantly alleviates inflammatory responses in DBA/1J mouse CIA model (Wang et al., 2017).In this study, CRME was orally administered once daily for 30 consecutive days (days 22–52 post-immunization) at three doses: 24.8, 49.7, and 99.4 mg/kg/day. Positive controls methotrexate (0.9 mg/kg/day) and Tripterygium glycosides (11.3 mg/kg/day) were given via the same route and schedule. The high-dose CRME group showed superior pharmacological activity to both controls across key endpoints: it reduced arthritis index scores from day 13, lowered hind paw swelling to 29.8% by day 51, mitigated body weight loss more effectively, and produced greater reductions in serum pro-inflammatory cytokines (TNF-α, IL-6, IL-1β, PGE_2_) and synovial NF-κB p65 expression (all *P* < 0.05; *P* < 0.001 for NF-κB p65). Histopathology confirmed less synovial hyperplasia, inflammatory cell infiltration, and capillary dilation in the high-dose CRME group. Regarding dose equivalency, CRME doses were selected based on its oral LD_50_ (4.6 g/kg in mice) and traditional usage, while positive control doses were converted from clinical human doses using standard body surface area scaling. Strict exposure-matching of active constituents was not performed, which is a limitation as pharmacokinetic differences may contribute to observed activity disparities. Future PK-PD studies will enable more rigorous potency comparisons between CRME and conventional anti-RA agents.

Mechanistically, the anti-inflammatory effects of CRME are primarily mediated through inhibition of the NF-κB, MAPK, and JAK/STAT signaling pathways. NF-κB appears to be a central target through which CRME exerts its anti-inflammatory activity (Uddandrao et al., 2025). In synovial tissues of CIA mice, CRME suppresses NF-κB p65 expression and concomitantly reduces serum levels of IL-1β, IL-6, TNF-α, and PGE_2_, thereby attenuating synovial inflammation and tissue damage (Lv S. W. et al., 2018). The MAPK pathway, including extracellular signal-regulated kinase (ERK), c-Jun N-terminal kinase (JNK), and p38 MAPK, interacts extensively with NF-κB to form a core signaling network governing synovial inflammation and hyperplasia in RA (Zhang et al., 2021). CRME also curtailed phosphorylation of ERK, JNK, and p38, which may jointly restrain pro-inflammatory mediator release and synovial hyperplasia (Lv et al., 2017b; Lv et al., 2025). In addition, CRME markedly suppresses the phosphorylation of JAK2 and STAT3 in synovial tissues of CIA animals (Lv et al., 2025).

**FIGURE 6:**
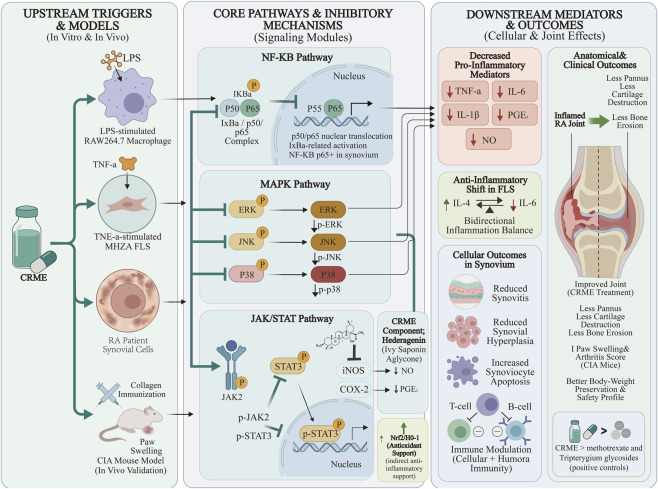
Mechanism of CRME’s anti-Inflammatory action in RA.

Notably, hederagenin, a representative triterpenoid saponin in CRME, reduces LPS-induced transcription of iNOS and COX-2 in a dose-dependent manner, and this is associated with decreased production of NO and PGE_2_ (Lee et al., 2015). Hederagenin may further mitigate inflammatory injury indirectly by engaging the Nrf2/HO-1 antioxidant defense program (Zhang et al., 2024). The integrated anti-inflammatory mechanisms of CRME in RA are summarized in Figure 6.

CRME exerts anti-RA effects through multiple *in vitro* (LPS-induced RAW264.7 macrophages, TNF-α-induced MH7A synovial cells, and RA patient-derived synovial cells) and *in vivo* (CIA mouse) models. It regulates the balance between pro-inflammatory mediators (TNF-α, IL-6, IL-1β, PGE_2_, NO) and anti-inflammatory cytokines (IL-4), inhibits hyperactivation of NF-κB, MAPK (ERK, JNK, p38) and JAK/STAT signaling pathways, and targets iNOS/COX-2 via its constituent hederagenin, with additional involvement of the Nrf2/HO-1 pathway.

### 7.2 Reversing synovial hyperplasia

**FIGURE 7:**
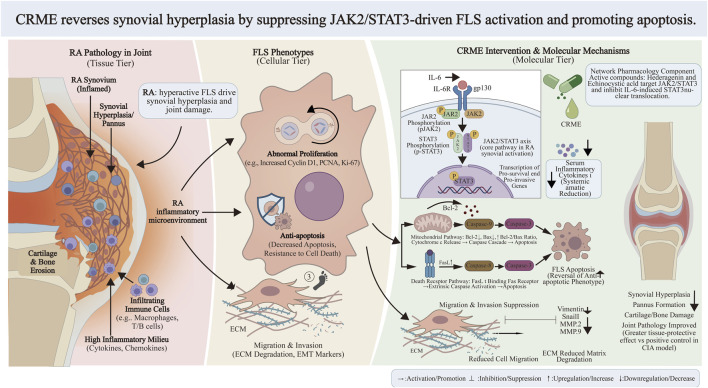
Schematic diagram illustrating the mechanism by which CRME reverses synovial hyperplasia in RA. (1) Suppressing JAK2/STAT3 pathway activation (reducing p-JAK2/JAK2 and p-STAT3/STAT3 ratios); (2) Inducing FLS apoptosis via mitochondrial (downregulating Bcl-2, upregulating Bax) and death receptor (increasing FasL) pathways; (3) Inhibiting FLS migration/invasion by downregulating Vimentin, Snail, MMP-2 and MMP-9; (4) Its active components (hederagenin, echinocystic acid) targeting JAK2/STAT3 to block IL-6-induced STAT3 nuclear translocation (network pharmacology evidence). Notably, CRME reduces synovial hyperplasia in CIA models with superior pharmacological activity over positive controls.

FLS in RA display aberrant proliferation, resistance to apoptosis, and increased invasiveness, which together contribute to synovial overgrowth and joint injury (Lee et al., 2013). Evidence suggests that CRME can counter these changes. Consistently, in CIA mice, CRME reduced synovial hyperplasia and mitigated tissue damage more effectively than the positive-drug control (Figure 7) (Wang et al., 2017).

Furthermore, the JAK/STAT cascade is a core signaling route underpinning aberrant synovial activation in RA. When dysregulated, it promotes FLS proliferation and contributes to disease progression (Malemud, 2018). *Caulophyllum robustum* shows remarkable efficacy in inhibiting JAK2 and STAT3 phosphorylation, significantly reducing the p-JAK2/JAK2 and p-STAT3/STAT3, while concurrently lowering serum inflammatory factor levels (Lv et al., 2025). CRME also counteracts the anti-apoptotic phenotype of FLS by engaging both mitochondrial and death receptor–mediated apoptotic programs. Specifically, it decreases Bcl-2, increases Bax, thereby reducing the Bcl-2/Bax ratio, and elevates FasL expression (Lv S. W. et al., 2018).

CRME also restrains FLS motility by reducing migratory and invasive behavior, accompanied by decreased expression of vimentin and Snail as well as MMP-2 and MMP-9 (Wang et al., 2017). Moreover, network-pharmacology analyses suggest that the saponins hederagenin and echinocystic acid may act on the JAK2/STAT3 axis and attenuate IL-6–triggered STAT3 nuclear translocation (Lv et al., 2025).

### 7.3 Inhibition of pannus formation

**FIGURE 8:**
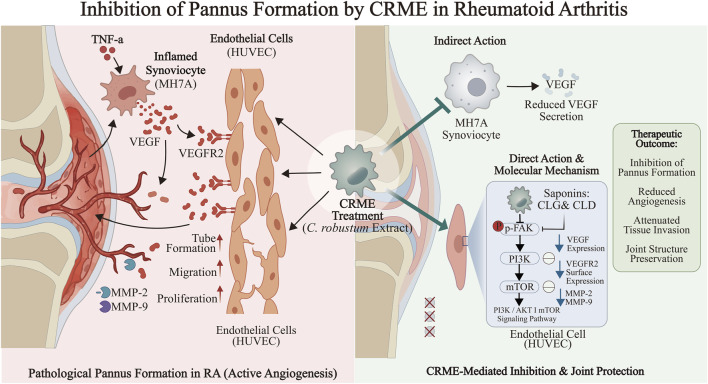
Schematic illustration of CRME-mediated inhibition of pannus formation in RA. (1) Indirect action: Reduces VEGF secretion from MH7A synoviocytes in a concentration-dependent manner; (2) Direct action and molecular mechanism: Its saponin components (CLG, CLD) suppress FAK phosphorylation, block the PI3K/AKT/mTOR pathway, and downregulate VEGF, VEGFR2, and MMP-2/9 expression, thereby inhibiting HUVEC proliferation/migration/tube formation. Therapeutic outcome: Inhibition of pannus formation, reduced angiogenesis, and joint structure preservation.

Pannus represents pathological vascularized connective tissue forming in RA, which is highly dependent on VEGF signaling pathways. This pathway underlies the formation of pathological structures that invade cartilage and bone (Leclair et al., 2022; Wang Y. et al., 2025). Studies confirm that CRME effectively inhibits this pathway, blocking pannus generation and development at multiple stages (Figure 8).

Mechanistically, CRME regulates synovial cells to reduce pro-angiogenic signals at their source. In the TNF-α-induced MH7A synoviocyte cell line, CRME at concentrations ranging from 100 to 500 μg/mL reduces VEGF secretion in a concentration-dependent fashion (Lv S. W. et al., 2018). Simultaneously, CRME acts directly on HUVEC, inhibiting their proliferation, migration, and *in vitro* tube formation capabilities. It reduces the protein expression of VEGF and its receptor VEGFR2, blocking angiogenesis at multiple stages (Cui M. H. et al., 2025). At the molecular level, the saponins CLG and CLD in *C. robustum* inhibit VEGF and MMP-2/9 expression by suppressing FAK phosphorylation, thereby interfering with PI3K/AKT/mTOR signaling (Cui M. H. et al., 2025).

In summary, CRME demonstrates potential for effectively inhibiting pannus formation and progression by directly suppressing endothelial cell function and indirectly reducing synovial cell-derived VEGF production, providing a mechanistic basis for its application in RA treatment.

### 7.4 Protection against bone destruction

**FIGURE 9:**
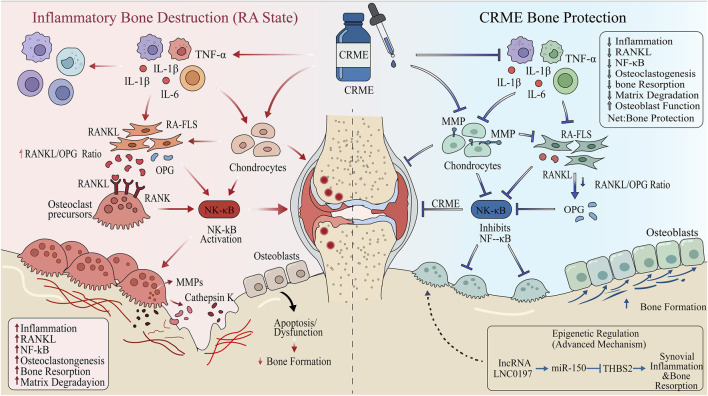
CRME mediates bone-protective mechanisms against inflammatory bone destruction in RA.

In advanced RA, chronic inflammation favors excessive osteoclast-driven resorption while osteoblast-mediated bone formation is impaired, producing a profound imbalance in bone remodeling that ultimately results in irreversible joint damage (Smolen, 2020). Accumulating evidence indicates that CRME exerts significant protective effects on the skeletal system. These effects appear to involve both direct regulation of key pathways governing bone metabolism and indirect improvement of the inflammatory bone microenvironment, as summarized in Figure 9.

Osteoclast formation and function are largely governed by the RANKL/RANK/OPG regulatory network (Abdulrahman et al., 2023). Pharmacological evidence indicates that CRME lowers RANKL expression and release in synovial fibroblasts, thereby shifting the local RANKL/OPG balance and limiting osteoclastogenesis within the joint milieu (Lv S. W. et al., 2018). In parallel, CRME suppresses NF-κB signaling, a pathway that is central to inflammatory responses and also contributes to RANKL-dependent osteoclast activation, and this inhibition is associated with reduced osteoclast differentiation and activity (Wu et al., 2017).

In addition to its actions on osteoclast-centered pathways, CRME may protect bone indirectly by curbing inflammatory pressure within the rheumatoid milieu. In experimental settings, CRME reduces the inflammatory cytokine burden, most notably TNF-α, IL-1β, and IL-6. Among these, IL-6 is a key driver of osteoclast formation and activation (Feng et al., 2022). TNF-α and IL-1β can further potentiate RANKL-dependent osteoclastogenic signaling and stimulate synovial cells as well as chondrocytes to increase matrix metalloproteinase (MMP) production, thereby accelerating breakdown of collagenous and other matrix components and weakening skeletal integrity (Hyc et al., 2016). By attenuating inflammatory signaling, CRME indirectly reduces MMP activity and osteoclast-stimulating cues, contributing to preservation of bone structural integrity.

Effective bone protection requires not only inhibition of bone resorption but also restoration of bone formation (Fensham et al., 2022). Persistent inflammation severely suppresses osteoblast function and promotes osteoblast apoptosis. By alleviating the inflammatory microenvironment, CRME provides more favorable conditions for osteoblast survival and activity. Moreover, CRME modulates signaling pathways such as NF-κB and MAPK, which are key regulators of osteoblast differentiation and function, suggesting a potential direct contribution to the restoration of bone formation.

Emerging evidence further suggests that CRME’s bone-protective effects may involve epigenetic regulatory networks. Long non-coding RNAs (lncRNAs), such as LINC01197, have been reported to influence synovial inflammation and bone resorption through the miR-150/THBS2 axis. Although direct evidence for *C. robustum* is currently limited, studies on related natural products, including salasinol and *Tripterygium wilfordii* extracts, indicate that modulation of such lncRNA-mediated pathways may represent an additional regulatory layer in inflammatory bone destruction (Cheng Y. et al., 2022; Miao et al., 2021; Piao et al., 2021; Yan et al., 2023).

CRME exerts bone-protective effects through (1) direct regulation of osteoclast-related pathways by inhibiting NF-κB activation and reducing RANKL secretion, thereby lowering the RANKL/OPG ratio and suppressing osteoclastogenesis; (2) indirectly improving the inflammatory milieu—for example, lowering TNF-α, IL-1β, and IL-6, which in turn reduces MMP-dependent matrix degradation; and (3) potential epigenetic regulation involving lncRNA-mediated signaling axes. These coordinated actions contribute to balanced bone metabolism, preservation of bone structural integrity, and overall protection against RA-associated bone destruction.

### 7.5 Immunomodulation

**FIGURE 10:**
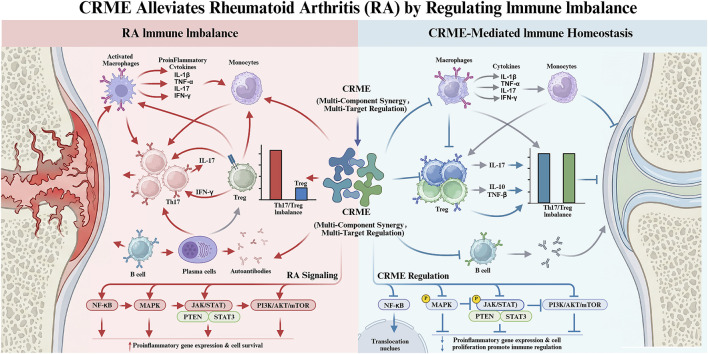
CRME alleviates RA by restoring immune homeostasis.

Immune dysregulation is a central pathological feature of RA, characterized by abnormal activation of innate, cellular, and humoral immune responses (Hoffmann et al., 2011). Pharmacological studies demonstrate that CRME exerts systemic immunomodulatory effects, correcting immune imbalance and promoting restoration of immune homeostasis. These effects primarily involve suppression of excessive innate immune activation and reestablishment of dynamic equilibrium between cellular and humoral immunity, as illustrated in Figure 10.

Aberrant activation of innate immune responses serves as both the initiator and a sustaining driver of RA-associated inflammation (Hong et al., 2024). CRME exhibits pronounced inhibitory effects on innate immunity. In adjuvant-induced arthritis (AA) rat models, CRME administered at doses of 17.31–69.23 mg kg^-1^ significantly reduces spleen indices, reflecting suppression of pathological immune organ hyperplasia. Functional immune assays—including carbon particle clearance, delayed-type hypersensitivity (DTH), and hemolysin production tests—demonstrate that *C. robustum* inhibits monocyte/macrophage phagocytic activity, suppresses DTH responses, modulates T-lymphocyte subsets, and reduces antibody production in immunized mice (Lv et al., 2017b). Consistently, CRME significantly lowers serum levels of pro-inflammatory cytokines such as IL-1β, TNF-α, IL-17, and IFN-γ in AA rats, thereby improving the pro-inflammatory immune microenvironment.

Imbalance between Th17 cells and Treg cells represents a key driver of immune dysregulation in RA (Fan et al., 2023). CRME displays robust regulatory effects on lymphocyte populations. It reduces the proportions of CD3^+^ and CD4^+^ T cells, increases CD8^+^ T cells, and markedly lowers the CD4^+^/CD8^+^ ratio (Lv et al., 2017b). In parallel, CRME significantly decreases Th1- and Th17-associated cytokines (IFN-γ and IL-17) while upregulating Th2- and regulatory cytokines, including IL-4, IL-10, and TGF-β (Lv et al., 2017b). Through this coordinated regulation of T-cell subsets and cytokine networks, CRME promotes restoration of Th17/Treg balance. Further studies indicate that triterpenoid saponins from *C. robustum* alleviate arthritis by correcting Treg/Th17 imbalance via modulation of the JAK/PTEN-STAT3 signaling pathway (Huang et al., 2023).

CRME also suppresses humoral immune responses, as evidenced by dose-dependent reductions in serum hemolysin levels and antibody production (Lv et al., 2017b). In CIA models, CRME not only alleviates arthritis symptoms but also significantly reduces spleen and thymus indices, supporting its systemic immunomodulatory effects (Lv et al., 2017b). Moreover, CRME demonstrates superior efficacy compared with positive control drugs in mitigating CIA-associated weight loss, suggesting a favorable safety profile (Wang et al., 2017). Notably, the combined administration of total saponins and total alkaloids further enhances the immunomodulatory activity of CRME, underscoring the importance of multi-component complementary interactions (Lv C. Y. et al., 2018).

Overall, CRME exerts comprehensive pharmacological effects in RA through coordinated regulation of multiple key signaling pathways, including NF-κB, MAPK, JAK/STAT, and PI3K/AKT/mTOR. These multi-component, multi-target actions suppress inflammatory responses, restore immune balance, inhibit synovial hyperplasia and pannus formation, and protect bone structure.

CRME exerts synergistic, multi-target immunomodulatory effects by (1) inhibiting macrophage overactivation and pro-inflammatory cytokine secretion; (2) restoring Th17/Treg balance through coordinated regulation of cytokines (decreased IL-17 and IFN-γ, increased IL-10 and TGF-β); (3) suppressing B-cell differentiation and autoantibody production; and (4) blocking hyperactivation of NF-κB, MAPK, JAK/STAT, and PI3K/AKT/mTOR signaling pathways. These integrated actions promote systemic immune homeostasis and alleviate RA-associated inflammatory responses.

## 8 Application of new technologies and research advances

The multi-target pharmacological mechanisms of *C. robustum* against RA have been increasingly elucidated through integrated computational and experimental approaches. Over the past 2 decades, advances in molecular docking, biomolecular interaction analysis, genomics, and metabolomics have provided important mechanistic insights into the molecular and cellular actions of its major active constituents.

### 8.1 Molecular docking and interaction technologies

Molecular docking and biomolecular interaction analysis are widely applied to identify potential targets and binding modes of natural products (Jeong et al., 2022; Patil and Patil, 2023). Docking approaches predict optimal ligand–target conformations and binding affinities *in silico* (Manju et al., 2017). Whereas experimental techniques such as surface plasmon resonance enable real-time, quantitative assessment of binding kinetics (Wang H. P. et al., 2025). Together, these complementary tools have become indispensable for mechanistic exploration of herbal medicines (Ahmad et al., 2024).

Using AutoDock virtual screening combined with Biacore T200 validation, hederagenin, echinocystic acid, and oleanolic acid from *C. robustum* were identified as inhibitors of histone deacetylases HDAC3 and HDAC8 (Zhao et al., 2020). Hederagenin exhibited dissociation constants of 2.96 μmol/L for HDAC3 and 0.94 μmol/L for HDAC8, indicating stronger binding affinity than the positive control, trichostatin A. Molecular simulations further revealed that hederagenin forms hydrogen bonds with ASP-225 and CYS-268 of HDAC3 and establishes hydrophobic interactions with LEU-323 and PRO-16 of HDAC8 (Zhao et al., 2020). In addition, docking analyses indicate that *C. robustum* alkaloids may act in a multi-target manner: caulophylline A and (+)-reticuline were predicted to interact with p38 MAPK and COX-2, respectively, with (+)-reticuline showing particularly strong affinity for COX-2. Caulophylline A displayed the highest binding score with p38 MAPK (Total Score = 8.2443) (Yuan, 2021). In addition, virtual screening identified 1-linolenoylglycerol and columbamine as potential ligands for phospholipase A_2_ (PLA_2_), with high binding scores (Yuan, 2021). These findings suggest that multiple constituents of *C. robustum* may modulate arachidonic acid metabolism, providing a molecular basis for its anti-inflammatory activity.

Recent studies have further highlighted the potential immunomodulatory relevance of saponins from *C. robustum*. CD_81_ has been validated as a therapeutic target for RA, as siRNA-mediated CD_81_ knockdown significantly ameliorated CIA in rats (Nakagawa et al., 2009). Molecular docking analyses indicate that certain saponins exhibit strong binding affinity toward the immune-related transmembrane protein CD_81_. Among them, Caulophyllum saponin B showed the strongest binding energy (−7.042 kcal/mol), suggesting stable interaction (Zhang et al., 2025). These findings imply that saponins from *C. robustum* may exert immunomodulatory effects in RA by targeting this validated pathogenic protein.

### 8.2 Metabolomics

Metabolomics offers a powerful systems-level approach for elucidating the holistic mechanisms of TCM by comprehensively profiling dynamic changes in endogenous small-molecule metabolites (Lin et al., 2019). Increasing evidence indicates that the anti-RA effects of *C. robustum* are closely associated with its capacity to reverse RA-related metabolic network disturbances and restore systemic metabolic homeostasis (Zhu et al., 2025).

In a CIA rat model, UPLC–Q-TOF/MS was employed to characterize endogenous urinary metabolites in normal, CIA, and CRME-treated groups. This analysis identified a panel of potential metabolic biomarkers associated with RA pathology and revealed that CRME significantly modulates metabolites including taurine, hippuric acid, phenylalanine, tyrosine, benzoic acid, pseudoephedrine, indole, indole-3-acetaldehyde, α-ketoglutaric acid, glycine, ornithine, lysine, methionine, and histidine. These metabolic alterations primarily involved pathways such as purine metabolism, pyrimidine metabolism, tryptophan metabolism, and the tricarboxylic acid (TCA) cycle (Lv et al., 2021).

Among the identified metabolites, taurine is widely recognized for antioxidant activity as well as anti-inflammatory and immunomodulatory effects (Fogagnolo et al., 2025; Lin et al., 2025). The marked changes in taurine levels observed in CIA rats suggest that CRME may alleviate RA pathology by regulating oxidative stress and inflammatory responses. Hippuric acid, a gut microbiota–derived metabolite of phenylalanine and tyrosine, reflects host–microbiota metabolic interactions. Alterations in hippuric acid levels are therefore closely linked to gut microbial homeostasis (Ermolenko et al., 2026). Regulation of hippuric acid by *C. robustum* may indirectly improve the intestinal microecological environment, thereby contributing to its therapeutic effects in RA.

Furthermore, phenylalanine, tyrosine, and other essential amino acids participate in multiple biosynthetic and signaling pathways, and their metabolic dysregulation has been associated with immune imbalance and chronic inflammation in RA (Muhammed et al., 2020). Modulation of amino acid metabolism by CRME may thus help restore metabolic equilibrium and attenuate inflammatory responses. At the pathway level, hyperactivation of purine and pyrimidine metabolism reflects abnormal proliferation and activation of immune cells and synovial fibroblasts in RA (Miao et al., 2017). Regulation of these pathways may suppress pathological cell proliferation and inflammatory mediator production, thereby contributing to immune tolerance and restoration of the Th17/Treg balance (Jiang et al., 2023).

Disruption of the TCA cycle, a central hub of cellular energy metabolism, indicates altered energy supply under RA pathological conditions (Auger et al., 2024; Lv et al., 2021). Restoration of TCA cycle function by CRME suggests normalization of cellular metabolic states and inhibition of aberrant cellular proliferation. Collectively, these metabolomic findings highlight the capacity of *C. robustum* to exert anti-RA effects through coordinated regulation of metabolic pathways and restoration of metabolic homeostasis.

### 8.3 Genomics

Genomics technologies enable the systematic interrogation of gene structure, function, and interaction networks at the whole-genome scale (Furlong, 2013). In contemporary TCM research, genomics-based approaches provide rigorous systems-level evidence for deciphering multi-target, multi-pathway pharmacological actions by capturing drug-induced reprogramming of global transcriptional landscapes (Dogiparthi et al., 2025). Notably, accumulating evidence links aberrant lncRNA expression and function to diverse human disorders, including malignancies, psychiatric diseases, and autoimmune conditions (Cui, 2023; Xiao et al., 2023). Mechanistically, sequence and higher-order structural alterations, dysregulated lncRNA transcription, and perturbed interactions with RNA-binding proteins can collectively reshape epigenetic states and thereby remodel gene expression programs (Miao et al., 2021). Against this backdrop, lncRNA–mRNA microarray profiling in a TNF-α–induced inflammatory L929 cell model demonstrated that CRME robustly counteracts inflammation-driven transcriptional dysregulation.

Transcriptome-wide analyses further indicate that CRME exerts anti-RA activity through coordinated, synergistic multi-target regulation. Specifically, 329 genes were significantly upregulated and 141 genes were downregulated between the CRME blank group and the model group; moreover, relative to the model group, CRME treatment yielded 409 differentially expressed genes, comprising 218 upregulated and 191 downregulated transcripts (Lv S. W. et al., 2020). Importantly, genes whose expression was reversed by CRME likely represent direct molecular nodes through which CRME mediates therapeutic effects. Gene Ontology enrichment implicated chromatin-centric processes—particularly nucleosome assembly and chromatin assembly/disassembly—as prominent biological themes, while molecular-function annotation highlighted the modulation of chemokine activity, binding, and downstream signal transduction. Consistently, KEGG pathway analysis mapped CRME-responsive genes to core inflammatory cascades, including NF-κB, MAPK, TNF, and Toll-like receptor signaling, and simultaneously enriched pathways related to autoimmune diseases such as RA and systemic lupus erythematosus. In parallel, CRME also intersected with signaling programs tightly coupled to RA progression, including VEGF, Wnt, and JAK–STAT pathways (Lv S. W. et al., 2020), collectively underscoring a network-level, multi-axis mode of action.

Quantitative real-time PCR corroborated the microarray results, confirming that CRME significantly upregulated Hist1h2ba, Egr1, and Ccl3 while downregulating Hist1h2bj, Zfp36, and Cxc12 (Lv S. W. et al., 2020). Given the established roles of these genes in histone architecture, inflammatory control, and immune-cell chemotaxis (Cheng T. T. et al., 2022). , their coordinated regulation provides mechanistic plausibility for CRME-mediated attenuation of RA-related inflammatory circuitry. Furthermore, construction of an lncRNA–mRNA weighted co-expression network revealed tight co-expression between these key mRNAs and multiple lncRNAs, suggesting that CRME may rewire downstream mRNA expression by modulating a set of regulatory lncRNAs, thereby forming an integrated gene regulatory circuitry that potentially operates at both transcriptional and epigenetic layers. In line with this, independent analyses reported that CRME markedly regulates multiple histone genes (e.g., HIST1H2BJ and HIST1H2BA), the zinc-finger protein ZFP36, chemokines CCL3 and CXCL2, and the early growth response factor EGR1 (Miao et al., 2021; Zhao et al., 2020). These transcripts occupy hub-like positions within the lncRNA–mRNA co-expression topology, supporting the notion that CRME may influence lncRNA dynamics to propagate regulatory effects onto target mRNAs. Collectively, this multi-tiered genomic network may converge to dampen inflammatory responses while restraining aberrant synoviocyte proliferation, providing a compelling systems-genomics rationale for CRME’s multi-target anti-RA pharmacology.

## 9 Discussion

Notably, the 2023 single-cell atlas of rheumatoid synovium has defined six cell-type abundance phenotypes that capture the pathological heterogeneity of RA, spanning lymphocyte-enriched to myeloid/fibroblast-dominant inflammatory subtypes (Zhang et al., 2023). The multi-target, multi-pathway pharmacological profile of *C. robustum* positions it to act across this spectrum of disease heterogeneity. Its inhibition of NF-κB and MAPK signaling directly targets the pro-inflammatory macrophage activity driving myeloid-dominant subtypes, while its modulation of JAK/STAT and PI3K/Akt pathways addresses the aberrant fibroblast proliferation and activation characteristic of fibroblast-enriched subtypes. For lymphocyte-rich subtypes, its ability to restore Th17/Treg balance and suppress effector T and B cell function targets the ectopic lymphoid activation that defines these pathotypes. While these mechanistic mappings are supported by robust preclinical evidence, their clinical relevance remains to be validated in human studies stratified by synovial inflammatory phenotypes.

Accumulating evidence indicates that *C. robustum* possesses considerable therapeutic potential in the management of RA. This review integrates current research on *C. robustum* from ethnopharmacological, phytochemical, pharmacokinetic, pharmacological, and systems-biology perspectives, providing a comprehensive overview of its anti-RA effects and underlying mechanisms. Overall, the available data support the notion that the anti-RA pharmacological activity of *C. robustum* arises from coordinated actions of multiple bioactive constituents rather than from a single dominant compound. Among these constituents, triterpenoid saponins represent the primary pharmacologically active class. Their biological activities and metabolic behaviors are strongly influenced by both AG structures and glycosylation patterns. Following oral administration, these saponins undergo biotransformation to yield more active AGs, such as hederagenin and caulophyllogenin, which contribute substantially to anti-inflammatory activity, inhibition of synovial hyperplasia, cartilage protection, suppression of osteoclast differentiation, and modulation of key molecular targets (Lv et al., 2019). In parallel, alkaloids constitute another important group of active compounds with reported anti-inflammatory, immunomodulatory, analgesic, neuroprotective, and anti-osteoclastogenic effects. Notably, increasing evidence suggests that these two classes of constituents act complementarily in the anti-RA process. In addition, several active components of *C. robustum* interact with P-gp, potentially enhancing intestinal absorption and improving overall bioavailability (Guo et al., 2020b).

Recent computational and experimental studies have further highlighted HDACs and the immune-related transmembrane protein CD_81_ as potential molecular targets of *C. robustum*. HDACs are increasingly recognized as key epigenetic regulators involved in synovial cell activation, inflammatory gene transcription, and fibroblast-like synoviocyte proliferation, making them attractive targets for RA intervention (Poon et al., 2024; Watson et al., 2024). Notably, recent work from our laboratory has resolved prior conflicting reports on HDAC activity in RA synovium by demonstrating isoform-specific dysregulation of HDAC family members (Sun and Lv, 2026). This isoform-specific regulation provides a strong rationale for developing targeted HDAC inhibitors, which aligns perfectly with our findings on the preferential HDAC3/8 inhibitory activity of *C. robustum* components. Similarly, CD_81_ plays a central role in immune cell activation and signaling (Duan and Hu, 2021)., and interactions between saponin components and CD_81_ may represent an additional pathway through which *C. robustum* modulates immune dysfunction in RA. Insights from metabolomic and genomic studies further support a systems-level mechanism of action. *Caulophyllum robustum* has been shown to partially reverse disturbances in purine/pyrimidine and tryptophan metabolism, while simultaneously modulating major signaling pathways, including NF-κB, MAPK, JAK–STAT, and PI3K/Akt/mTOR. Through these integrated regulatory effects, *C. robustum* contributes to restoration of Th17/Treg balance and attenuation of chronic inflammatory responses, underscoring its multi-dimensional and multi-pathway therapeutic profile.

Despite these advances, several critical challenges remain to be addressed. Notably, all currently available pharmacological evidence for *C. robustum* is derived from preclinical studies While extensive pharmacokinetic and metabolic studies in rats have clarified the absorption, distribution, metabolism, and excretion profiles of its major bioactive saponins, no human pharmacokinetic data or clinical trials have been completed to date. While traditional usage provides empirical safety signals, formal first-in-human assessments are required to establish human safety, tolerability, and pharmacokinetics, followed by stratified clinical trials to validate efficacy across RA inflammatory subtypes. From a regulatory perspective, the development of *C. robustum* as a phytomedicine will need to align with international guidelines for TCM/herbal drug development, balancing traditional multi-component complexity with modern regulatory requirements for clinical evidence and quality consistency. Systematic toxicological profiling remains incomplete. Data on acute, chronic toxicity, hepatotoxicity/nephrotoxicity risk, and reproductive toxicity are currently lacking, which are essential for defining the clinical safe dose range and identifying potential toxic components.

Furthermore, standardization of herbal preparations represents a key bottleneck. While preliminary studies have identified candidate marker compounds, including representative triterpenoid saponins (Cauloside G, Cauloside H, Leonticin D, Cauloside D, Cauloside B, Cauloside C) and alkaloids, and developed analytical methods such as quantitative analysis of multi-components by single-marker and UPLC-MS/MS for their quantification, these laboratory-specific protocols have not yet been standardized into universal industry standards. Notably, analysis of 17 batches of samples from 8 geographical origins revealed substantial variations in constituent profiles, highlighting the critical impact of geographical origin, harvesting time, and processing methods on phytochemical consistency (Guo et al., 2017). Establishing a harmonized quality control system to ensure batch-to-batch reproducibility is therefore a prerequisite for its clinical translation. In addition, the continued discovery of new constituents suggests that further bioactive compounds remain to be identified. Safety and toxicity profiles have not yet been comprehensively established, and the poor intestinal permeability and low oral bioavailability of saponins present formulation challenges. Moreover, whether the synergistic interactions between alkaloids and saponins extend to absorption, metabolism, and pharmacokinetic processes remains to be clarified.

## 10 Future perspectives

In response to the aforementioned unresolved issues, this review proposes the following future research directions:

1. Verification of complementary Mechanisms: The interaction network between triterpenoid saponins and alkaloids across ADME should be elucidated. It should also be determined whether alkaloids modulate saponin interconversion and bioavailability by regulating the gut microbiota and metabolic enzyme systems. The molecular basis underlying complementary enhancement should then be validated using a systems pharmacology framework. In addition, comparative evaluation of different saponin–alkaloid combinations is warranted to clarify whether synergy depends on specific saponin sugar-chain architectures or on the core alkaloid scaffold.
2. Formulation Optimization: Develop nanocarrier systems such as liposomes, micelles, and nanoparticles to enhance the solubility and permeability of saponins, thereby improving bioavailability. Recent studies have confirmed that nanocarrier-delivered triterpenoid saponins, such as ginsenoside-based nanocarrier drug delivery systems, can enhance oral bioavailability by 11.8 times (Nguyen et al., 2024).
3. Target Mechanism Validation: Conduct *in vivo* and *in vitro* experiments to elucidate the pharmacological mechanisms by which HDAC3 and CD_81_ mediate the anti-RA effects of *C. robustum*, with a focus on the promising bioactive compounds hederagenin, echinocystic acid, and CD_81_-targeting saponins.
4. Clinical Translational Research (Top Priority): First, conduct first-in-human studies to evaluate the safety, tolerability, and pharmacokinetic profile of *C. robustum* in healthy volunteers, as no human pharmacokinetic data currently exists. Subsequently, perform well-designed phase II and large-scale phase III clinical trials to systematically evaluate the therapeutic efficacy of *C. robustum* for RA, with long-term follow-up to confirm sustained benefits and long-term safety.
5. Comprehensive Safety Assessment: Systematically conduct preclinical toxicological studies, including acute/chronic toxicity, hepatotoxicity/nephrotoxicity evaluation, and reproductive toxicity assays, to fully characterize the safety profile, identify potential toxic components, and establish a well-defined safe dose range for clinical translation.
6. Standardized Quality Control System Development: Building on existing analytical methods for saponin quantification, establish harmonized quality control protocols, including defining core marker compounds, validating standardized HPLC/LC-MS/MS analytical platforms, and systematically evaluating the impact of geographical origin and harvesting conditions on constituent profiles, to ensure batch-to-batch consistency for clinical trials and future commercialization.
7. Novel Compound Discovery: Employing advanced analytical techniques such as cryogenic chromatography to achieve the separation and identification of unstable compounds, and experimentally verifying their biological activities, including anti-RA effects.

As with other anti-RA plant extracts, the journey of *C. robustum* from lab to market benefits from its well-documented traditional safety and multi-target regulatory advantages, while facing common challenges of standardized quality control and translational clinical validation. In summary, based on current preclinical evidence, *C. robustum* holds potential as an ethnomedicinal resource, and represents a promising candidate for further anti-RA therapy development, with subsequent translational and clinical studies needed to validate its clinical application. By integrating traditional knowledge with modern pharmacological and systems-biology approaches, continued investigation of *C. robustum* may not only support its development as a therapeutic agent for RA but also provide broader insights into the rational modernization of multi-component traditional medicines.
